# Fluid–Structure Interaction Models of Bioprosthetic Heart Valve Dynamics in an Experimental Pulse Duplicator

**DOI:** 10.1007/s10439-020-02466-4

**Published:** 2020-02-07

**Authors:** Jae H. Lee, Alex D. Rygg, Ebrahim M. Kolahdouz, Simone Rossi, Stephen M. Retta, Nandini Duraiswamy, Lawrence N. Scotten, Brent A. Craven, Boyce E. Griffith

**Affiliations:** 1 Department of Mathematics, University of North Carolina, Chapel Hill, NC USA; 2 Division of Applied Mechanics, Office of Science and Engineering Laboratories, Center for Devices and Radiological Health, United States Food and Drug Administration, Silver Spring, MD USA; 3 Victoria, BC Canada; 4 Departments of Mathematics, Applied Physical Sciences, and Biomedical Engineering, University of North Carolina, Chapel Hill, NC USA; 5 Carolina Center for Interdisciplinary Applied Mathematics, University of North Carolina, Chapel Hill, NC USA; 6 Computational Medicine Program, University of North Carolina, Chapel Hill, NC USA; 7 McAllister Heart Institute, University of North Carolina, Chapel Hill, NC USA

**Keywords:** Immersed boundary method, Finite element method, Porcine aortic valve, Bovine pericardial valve

## Abstract

Computer modeling and simulation is a powerful tool for assessing the performance of medical devices such as bioprosthetic heart valves (BHVs) that promises to accelerate device design and regulation. This study describes work to develop dynamic computer models of BHVs in the aortic test section of an experimental pulse-duplicator platform that is used in academia, industry, and regulatory agencies to assess BHV performance. These computational models are based on a hyperelastic finite element extension of the immersed boundary method for fluid–structure interaction (FSI). We focus on porcine tissue and bovine pericardial BHVs, which are commonly used in surgical valve replacement. We compare our numerical simulations to experimental data from two similar pulse duplicators, including a commercial ViVitro system and a custom platform related to the ViVitro pulse duplicator. Excellent agreement is demonstrated between the computational and experimental results for bulk flow rates, pressures, valve open areas, and the timing of valve opening and closure in conditions commonly used to assess BHV performance. In addition, reasonable agreement is demonstrated for quantitative measures of leaflet kinematics under these same conditions. This work represents a step towards the experimental validation of this FSI modeling platform for evaluating BHVs.

**Electronic supplementary material:**

The online version of this article (10.1007/s10439-020-02466-4) contains supplementary material, which is available to authorized users.

## Introduction

Worldwide, nearly 300,000 aortic valve replacements are performed annually, and the rate of heart valve replacement is projected to exceed 850,000/year by 2050.^16^ Bioprosthetic heart valves (BHVs) are commonly constructed from fixed porcine heart valves or bovine or porcine pericardial tissues. BHVs generate flow patterns that mimic those of the normal human aortic valve and typically allow patients to be managed without chronic anticoagulation.^58^ Unfortunately, BHVs have a limited durability, and they typically fail 10–15 years post implantation, primarily from tissue degeneration or calcification.^61^

Computer modeling and simulation (CM&S) can be used throughout the life cycle of prosthetic valve design and regulatory approval. CM&S is a cost- and time-efficient complement to traditional bench testing that can assess device performance under a broader range of conditions than those listed in the instructions for use, including patient-specific conditions.^46^ Simulations may be used in the design phase to optimize device design. Credible simulation data may also be leveraged in regulatory applications to support claims of device safety and effectiveness. Indeed, using modeling and simulation to support regulatory decision-making is a strategic priority area for the U.S. FDA Center for Devices and Radiological Health.^54^ CM&S is well-suited for performing root cause analyses to understand impaired device function. For example, CM&S provides information about valve performance that is difficult to acquire on the bench, including assessing the impact of non-circular configurations on transcatheter BHVs.^21^ To realize its full impact in device regulation, however, CM&S results must be shown to be credible through verification and validation (V&V).^3^

A fluid–structure interaction (FSI) approach is necessary to model heart valves across the full cardiac cycle.^73^ Accounting for coupling between the flexible valve leaflets and the fluid flow is crucial in studying the effect of vortices in the aortic sinuses, predicting fluid-induced shear stress on the leaflets, and assessing valve performance by quantifying the valve orifice area and regurgitation.^74^ A widely used approach to simulating cardiovascular FSI is the arbitrary Lagrangian–Eulerian (ALE) method,^5^,^64^ which uses body-conforming meshes for the fluid and solid. ALE methods have realized limited success in simulating the dynamics of heart valves to date, however, because of the substantial challenges posed by dynamically generating geometrically conforming discretizations of thin structures that undergo substantial motion.^5^,^64^

Non-body conforming discretizations, which avoid the difficulties of body-fitted grids, are now widely used to model heart valve dynamics. One of the earliest of these types of approaches is the immersed boundary (IB) method,^50^ which was introduced by Peskin to simulate heart valves.^48^,^49^ The IB formulation allows the structural discretization to be independent of the fluid grid and thereby facilitates models with very large structural deformations.^30^ Extensions to the IB method have also been used to simulate heart valves,^26^,^29^ but in most cases, these prior simulations were not fully resolved, and the valve leaflets were described using only simple structural models based on linear elasticity. Other studies^9^,^25^ used anisotropic models of native and bioprosthetic valves but did not address the diastolic phase of the cardiac cycle. Flamini *et al*.^22^ used an IB approach to simulate aortic valve FSI across multiple cardiac cycles, but they used a simplified description of the aortic valve mechanics. Hasan *et al*.^31^ also used an IB approach to simulate FSI in a subject-specific aortic root model, and they used realistic hyperelastic constitutive models to describe the valve leaflets, but their model has not been validated. In addition, neither study used subject-specific driving or loading conditions.

Methods also have been developed that combine features of ALE and IB-like approaches, including the hybrid fictitious domain/ALE method^17^ and the immersogeometric (IMGA) method.^34^,^38^,^71^,^72^,^74^ These methods also seek to relax the need to use body-conforming discretizations. Prior models of aortic valves using a fictitious domain/ALE method^17^ showed instabilities when simulated under physiological Reynolds numbers and transvalvular pressures. The IMGA method has been used in several studies on pericardial BHVs that include experimentally derived constitutive models of the valve leaflets. Hsu *et al*.^34^ validated the leaflet kinematics of their model by comparing the cross-sectional profiles of the leaflets to those from dynamic *in vitro* experimental measurements.^36^ Xu *et al*.^72^ used *in vivo* imaging data to drive their simulations and compared the fluid flow patterns with those from magnetic resonance imaging. These models both employed isotropic descriptions of the pericardial BHV leaflets. Wu *et al*.^71^ used an anisotropic model that incorporated the fiber structure of a bovine pericardial valve that was developed and validated with experimental data from Sun and Sacks.^66^ These prior studies did not use boundary condition models that established flow conditions directly comparable to available experimental or clinical data.

Other methods that avoid body-conforming discretizations include smoothed particle hydrodynamics (SPH).^42^ The SPH approach,^42^ however, cannot fully impose incompressibility, and prescribing boundary conditions is difficult. In addition, several studies have used cut-cell-like methods^12^,^13^,^39^ to simulate valve FSI, but these studies also used linearly elastic leaflet models and have not yet been validated.

To date, there have been relatively few experimental validation studies of FSI models of native or bioprosthetic aortic valves.^62^,^67^ The two studies by Tang *et al*.^67^ and Sigüenza *et al*.^62^ use idealized isotropic leaflet models that do not account for leaflet anisotropy and, as a result, their simulations show discrepancies in the dynamics of the valve compared with experimental data. These studies also do not use flow domains that are long enough to allow the complex flow patterns downstream of the valve to develop fully. The model of Sigüenza *et al*. exhibits incomplete closure and experiences substantial regurgitation during diastole, leading to an underestimation of the transvalvular pressure gradient. The study of Sigüenza *et al*. also uses flow rate boundary conditions throughout the cycle, which introduces bias in the valve dynamics.

This study develops a computational FSI model based on the IB method of an experimental pulse-duplicator platform for simulating BHV dynamics. The model is calibrated using relatively limited experimental data, and this study describes initial work towards the V&V of this model for porcine tissue and bovine pericardial BHVs. Our models of the leaflet mechanics are based on experimental tensile test data^7^,^8^,^40^ of fixed tissues that are similar to the biomaterials used to construct porcine aortic and bovine pericardial BHVs. To provide realistic driving and loading conditions across the full cardiac cycle, we use reduced-order models that are calibrated using pressure and flow data acquired from the pulse-duplicator systems. Simulation results are compared to experimental data obtained from a commercial ViVitro pulse duplicator and a customized experimental apparatus based on the ViVitro pulse duplicator.

## Materials and Methods

### Experimental Pulse Duplicator

**Figure 1:**
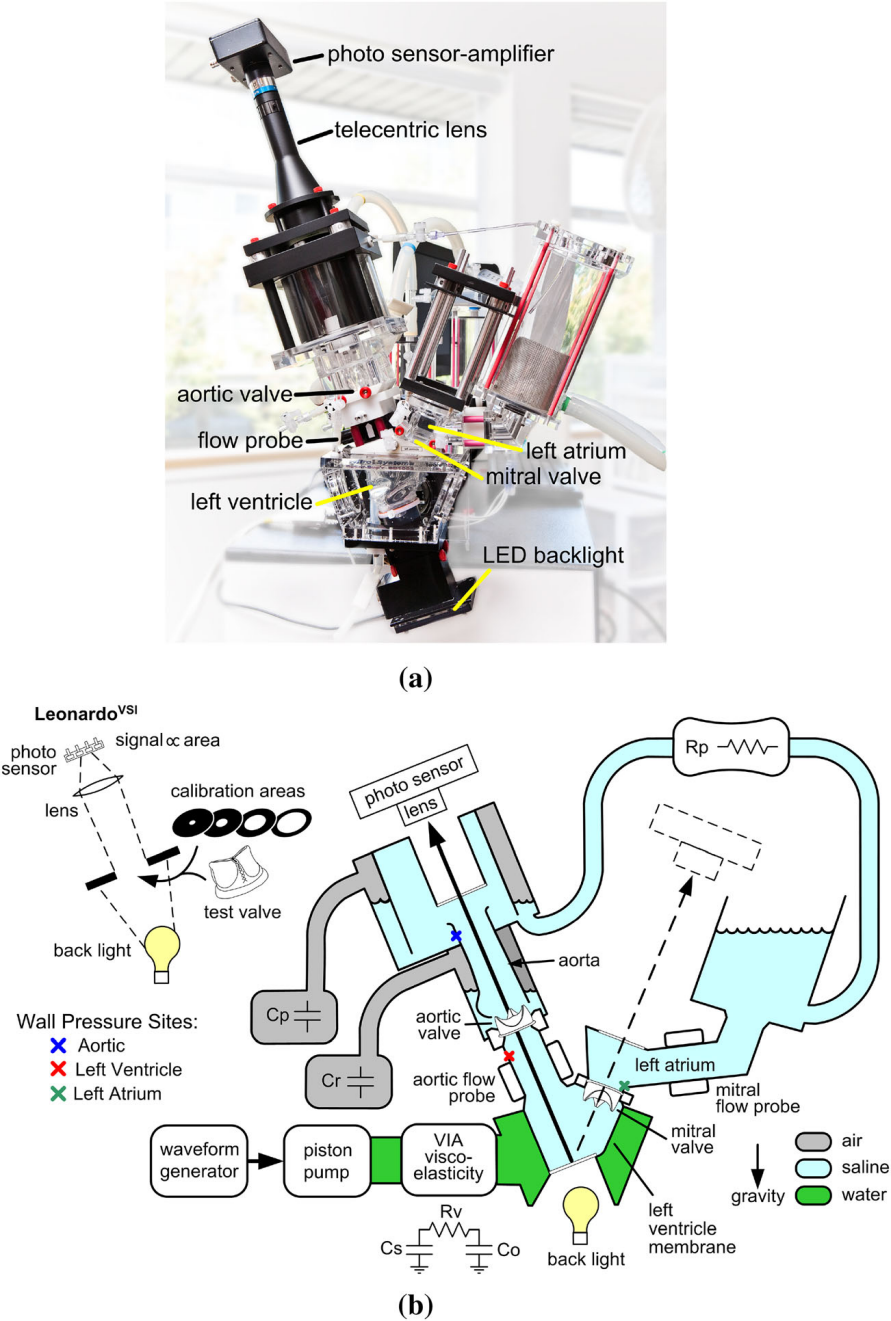
(a) A customized pulse duplicator based on the commercial ViVitro pulse-duplicator system adapted with prototype electro-optical subsystem for measuring aortic valve projected dynamic valve area (PDVA), or alternate configuration for measuring mitral valve PDVA. (b) A schematic diagram of the custom pulse duplicator based on the commercial ViVitro pulse-duplicator system adapted with prototype electro-optical subsystem for measuring aortic valve PDVA or alternate configuration for measuring mitral valve PDVA. The commercial ViVitro system is similar but lacks the back light and the photo sensor for acquiring PDVA.

*In vitro* experiments are performed using two different experimental platforms, including a ViVitro Pulse Duplicator (ViVitro Labs, Inc., Victoria, BC, Canada) available through the FDA Cardiac Device Flow Lab and a customized pulse duplicator developed by Scotten that is similar to the commercial ViVitro system. Figure 1a details Scotten’s customized system. It includes a prototype *Leonardo* electro-optical sub-system^60^ to assess projected dynamic valve area (PDVA). The commercial ViVitro system is similar but uses high-speed videography to assess valve kinematics, from which we reconstruct PDVA data *via* automatic image analysis in DataTank (Visual Data Tools, Inc., Chapel Hill, NC, USA). We also measure flow rates and pressures, as indicated in Fig. 1b. Experiments in the customized pulse duplicator use a 25 mm Labcor TLBP A Supra (Labcor Laboratórios Ltd., Belo Horizonte, Brazil) porcine aortic valve. Flow and pressure signals are filtered at 100 Hz, and PDVA signals are not filtered. Experiments at the FDA use a Model 2800 25 mm Carpentier–Edwards PERIMOUNT RSR (Edwards Lifesciences, Irvine, CA, USA) bovine pericardial aortic valve. Flow signals are filtered at 100 Hz, and pressure signals are not filtered. Both experiments use saline as the test fluid for this initial study because the viscosities of common blood analogues are more sensitive to temperature than saline. In addition, saline is widely used for hydrodynamic assessments of heart valves.^56^ For instance, the ISO 5840-3 standard allows saline to be used as a test fluid,^11^ and the most recent inter-laboratory study by Wu *et al*.^70^ also used saline as the test fluid. Results presented in Supplemental Materials Section G examine the effect of using a Newtonian blood analogue instead of saline in the computational model.

### Solid Mechanics Models

#### Leaflet Mechanics

We describe the biomechanics of the valve leaflets using the framework of nonlinear solid mechanics.^32^ Briefly, leaflet deformations are described by the mapping ${\varvec{\chi }}={\varvec{\chi }}({\varvec{X}},t)$ between reference coordinates ${\varvec{X}}$ and current coordinates ${\varvec{x}}$ at time *t*. The valve leaflets are treated as anisotropic, incompressible, hyperelastic materials. For a hyperelastic material, the first Piola–Kirchhoff stress ${\mathbb {P}}$ is related to a strain-energy functional $\Psi ({\mathbb {F}})$*via*^31^

1 $$ {\mathbb {P}}= \frac{\partial \Psi }{\partial {\mathbb {F}}},$$

in which ${\mathbb {F}}= \partial {\varvec{\chi }}/\partial {\varvec{X}}$ is the deformation gradient tensor. We split the strain energy functional into isochoric and volumetric parts,

2 $$ \Psi ({\mathbb {F}}) = W({\overline{{\mathbb {F}}}}) + U(J), $$

in which ${\overline{{\mathbb {F}}}} = J^{-1/3}{\mathbb {F}}$ and $J = \det {{\mathbb {F}}}.$ We use a modified version of the Holzapfel–Gasser–Ogden model from Murdock *et al*.^47^ with the addition of angle dispersion.^24^ The isochoric part of the model, $W(\overline{{\mathbb {F}}}),$ includes an isotropic contribution from the extracellular matrix and an anisotropic contribution from the collagen fibers embedded in the leaflets,

3 $$ W({\overline{{\mathbb {F}}})} = W_{{\text {iso}}}({\overline{{\mathbb {F}}}}) + W_{{\text {aniso}}}({\overline{{\mathbb {F}}}}). $$

**Figure 2:**
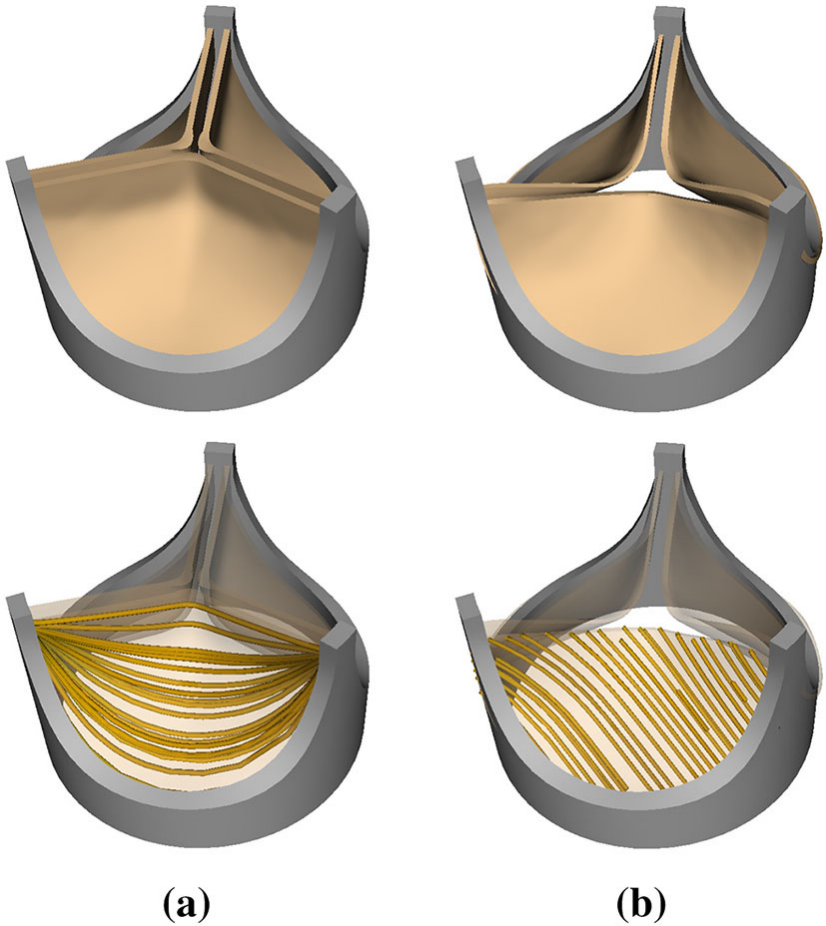
(a) Model porcine bioprosthetic valve geometry and fiber architecture. The idealized geometry of the Labcor TLBP A Supra porcine aortic valve is reconstructed based on literature values.^20^ The model fiber structure is generated using Poisson interpolation.^69^ (b) Model bovine pericardial bioprosthetic valve geometry and fiber architecture. This valve geometry is obtained from micro-CT imaging of a Carpentier–Edwards PERIMOUNT RSR Model 2800 surgical aortic heart valve. The model fiber structure is generated based on the small angle light scattering (SALS) data of Sun *et al*.^66^ The SALS data show that the mean fiber orientation of a bovine pericardial valve leaflet is $45^{\circ }$.

This model describes the extracellular matrix as an exponential neo-Hookean material along with a collagen fiber reinforcement model accounting for angle dispersion,

4 $$ W_{{\text {iso}}}({\overline{{\mathbb {F}}}})= C_{10}\{\exp {\left[ C_{01}({\bar{I}}_1-3)\right] }-1\}, $$

5 $$ W_{\text{aniso}} ({\overline{{\mathbb {F}}}})= \frac{k_{1}}{2k_{2}}\left\{\exp {\left[ k_{2}(\kappa {\bar{I}}_{1} + (1-3\kappa ){\bar{I}}_{4}^{\star }-1)^{2}\right] -1}\right\}, $$

in which $${\bar{I}}_{1} = {\text {tr}}({\overline{{\mathbb {C}}}})$$ is the first invariant of the modified right Cauchy–Green strain tensor $${\overline{{\mathbb {C}}}} ={\overline{{\mathbb {F}}}}^{\text {T}}{\overline{{\mathbb {F}}}},$$$${\bar{I}}_{4}^{\star } =\max ({\bar{I}}_{4}, 1) = \max ({\varvec{e}}_{0}^{\text {T}} {\bar{{\mathbb {C}}}}{\varvec{e}}_{0}, 1),$$ and $${\varvec{e}}_{0}$$ is a unit vector aligned with the mean fiber direction in the reference configuration. By construction, $${\bar{I}}_{4}^{\star }$$ is nonzero in extension but not in compression. The parameter $$\kappa \in \left[ 0,\frac{1}{3}\right] $$ describes collagen fiber angle dispersion. If $$\kappa = 0,$$ the fibers are perfectly aligned and the constitutive model becomes the same as that of Murdock *et al*.^47^ By contrast, if $$\kappa = \frac{1}{3},$$ then the model describes an isotropic distribution of fibers, and we obtain an isotropic model with no preferred direction of fiber reinforcement. The parameters for the porcine aortic valve are fit to experimental tensile test data from Billiar and Sacks^7^,^8^ for glutaraldehyde-fixed porcine aortic valves (Fig. 2a). We obtain $$C_{10} = 0.302\, {\text {kPa}},$$$$C_{01} = 3.25,$$$$k_{1} = 0.197\,{\text {MPa}},$$$$k_{2} =0.001,$$ and $$\kappa = 0.0.$$ The parameters for the bovine pericardial valve are fit to biaxial data from Kim *et al*.,^40^ and we obtain $$C_{10} = 0.119\,{\text {kPa}},$$$$C_{01} =22.59,$$$$k_{1} = 2.38\,{\text {MPa}},$$$$k_{2} = 149.8,$$ and $$\kappa = 0.292.$$ For further details, see Supplemental Materials Section B. The mathematical framework used in this study treats the leaflets as exactly incompressible. Thus, within our numerical framework, the volumetric part of the strain energy,

6 $$ U(J) = \beta (J\ln {J} - J + 1), $$

can be viewed as a stabilization term. For further details, see the summary of the study of Vadala-Roth *et al*.^68^ in Supplemental Materials Section E. We use $\beta =14.1\,{\text {MPa}}$ in our simulations.

The geometry of the Labcor TLBP A Supra porcine aortic valve is constructed based on literature values.^20^ A model collagen fiber architecture is created using Poisson interpolation^69^ (Fig. 2a). The pericardial valve geometry is reconstructed from micro-CT images of a Carpentier–Edwards PERIMOUNT RSR Model 2800 surgical aortic heart valve (Fig. 2b). The mean fiber orientation is chosen to be $45^{\circ }$ (Fig. 2b), following the small angle light scattering data of Sun *et al*.^66^

#### Aortic Test Section

The wall of the aortic test section is glass. To avoid the expensive linear solvers required by exactly imposing the rigidity constraint within the present computational framework,^37^ we instead use a penalty method that models the test section as a stiff neo-Hookean material with^31^

7 $$ W_{{\text {wall}}} = \frac{c_{{\text {wall}}}}{2}({\bar{I}}_{1} - 3). $$

Additional structural forces are included in the rigid test section model,

8 $$ {\varvec{F}}({\varvec{X}},t) = \kappa _{{\text {wall}}}({\varvec{X}}- {\varvec{\chi }}({\varvec{X}},t)). $$

In the limit as $c_{{\text {wall}}}$ and $\kappa _{{\text {wall}}}$ become large, the test section becomes effectively rigid and stationary. We use $c_{{\text {wall}}} = 33.1\,{\text {kPa}}$ and $\kappa _{{\text {wall}}} =852\,{\text {MPa cm}}^{-2}.$

### Fluid Model and Boundary Conditions

We use the incompressible Navier–Stokes equations to model the test fluid in the aortic test section of the pulse duplicator as a viscous incompressible fluid. We model the saline solution with a uniform density $\rho = 1.0\,{\text {g cm}}^{-3}$ and a uniform dynamic viscosity $\mu = 1.0\,{\text {cP}}$ (saline at $25\,^{\circ }{\text {C}}$) for both cases. Results from additional simulations using a blood analogue fluid with density $1.0\,{\text {g cm}}^{-3}$ and viscosity $3.5\,{\text {cP}}$ are provided in Supplemental Materials Section G. These simulations indicate that the large-scale flow structures and leaflet kinematics are similar between saline and the blood analogue.

**Figure 3:**
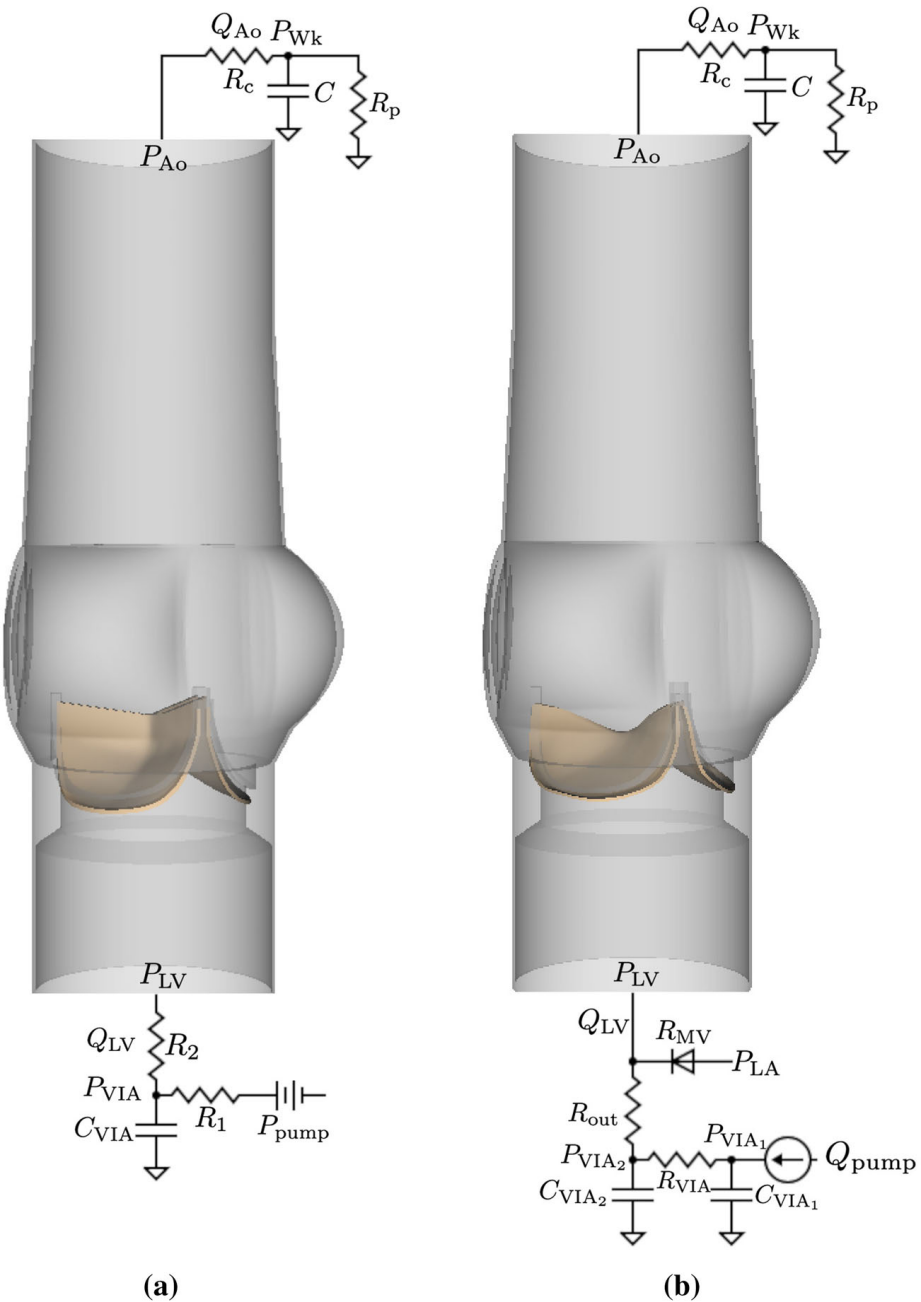
Three-dimensional aortic test section models for the porcine (a) and bovine pericardial (b) BHV simulations along with the reduced-order models that provide driving and loading conditions. Three-element Windkessel models are used at the downstream (outlet) for both cases. (a) A three-element Windkessel model is used at the upstream (inlet) for the porcine aortic valve simulations. The pump pressure is derived from pressure and flow data from the ventricular outflow tract of the pulse duplicator. (b) Because the pump flow waveform and atrial pressure are available for the bovine pericardial valve experiments, a more detailed pump model is used upstream for the bovine pericardial valve simulations.

Three-element Windkessel (R–C–R) models establish downstream loading conditions for the aortic test section for both cases (Fig. 3). A three-element Windkessel model is also used for the porcine BHV simulations to capture the upstream driving conditions for the aortic test section (Fig. 3a). Additional data are available for the bovine pericardial case, including the experimental pump flow and atrial pressure waveforms, which allow for a more complete description of the upstream components of the system (Fig. 3b). In both cases, we impose a combination of normal traction and zero tangential velocity boundary conditions at the inlet and outlet of the FSI model to couple the reduced-order models to the detailed description of the flow within the aortic test section. The values of the resistances and compliance for the upstream model are $$C_{{\text {VIA}}} =0.1\,{\text {mmHg mL}}^{-1},$$$$R_{1} = 0.15\,{\text {mmHg mL}}^{-1}\,{\text {s}},$$ and $$R_{2} = 0.15\,{\text {mmHg mL}}^{-1}\,{\text {s}}$$ for the porcine aortic valve case, which characterize compliance and resistance of the VIA system. The values for the bovine pericardial valve case are $$C_{{\text {VIA}}_{1}} = 0.0275\,{\text {mmHg mL}}^{-1},$$$$C_{{\text {VIA}}_{2}} = 0.0347\,{\text {mmHg mL}}^{-1},$$$$R_{{\text {VIA}}} =0.15\,{\text {mmHg mL}}^{-1}\,{\text {s}},$$ and $$R_{{\text {out}}} =0.0898\,{\text {mmHg mL}}^{-1}\,{\text {s}}.$$ The mitral valve is modeled as a diode, with a resistance of $$R_{{\text {MV}}} = 0.0280 \,{\text {mmHg mL}}^{-1}\,{\text {s}}$$ when the valve is open. The values for the downstream model are $$R_{{\text {c}}} = 0.0218 \,{\text {mmHg mL}}^{-1}\,{\text {s}},$$$$R_{{\text {p}}} = 1.31 \,{\text {mmHg mL}}^{-1}\,{\text {s}},$$ and $$C = 0.915\,{\text {mmHg mL}}^{-1}$$ for the porcine BHV, and $$R_{{\text {c}}} = 0.0282\,{\text {mmHg mL}}^{-1}\,{\text {s}},$$$$R_{{\text {p}}} = 1.22 \,{\text {mmHg mL}}^{-1}\,{\text {s}},$$ and $$C = 1.27\,{\text {mmHg mL}}^{-1}$$ for the bovine pericardial BHV. Supplemental Materials Section C provides additional details. We use solid wall (zero velocity) boundary conditions on the remainder of the boundaries in the computational domain of the FSI model.

### Fluid–Structure Interaction

We use a finite element (FE) extension of the IB method^28^ to simulate FSI. Supplemental Materials Section D provides details on the continuum formulation and numerical approximations. Reduced-order boundary condition models are coupled to the FSI model following the approach detailed by Griffith *et al*.^29^

### Numerical Discretizations

Simulations are performed using a geometrically realistic model of the aortic test section of the pulse duplicator with length 10.1 cm and diameter $28\,{\text {mm}}$ (see Fig. 3). The aortic test section is embedded in a square computational domain with side lengths of 10.1 cm. In our IB formulation, Eulerian variables are approximated using a block-structured adaptively refined Cartesian grid, and Lagrangian variables are approximated using an unstructured FE mesh that conforms to the geometry of the structure.^28^ The effective fine-grid resolution of the Cartesian grid is approximately $0.39\,{\text {mm}}.$ The structural meshes use second-order hexahedral (27-point) elements for the BHV leaflets and first-order tetrahedral (4-point) elements for the aortic test section. The aortic test section and porcine BHV meshes were generated using Trelis (Computational Simulation Software, LLC, American Fork, UT, USA), and the bovine pericardial BHV mesh was generated using Pointwise (Pointwise, Inc., Ft. Worth, TX, USA). The average grid-spacings of the meshes are $0.4\,{\text {mm}}$ for the aortic test section, $0.59\,{\text {mm}}$ for the porcine aortic valve, and $0.75\,{\text {mm}}$ for the bovine pericardial valve. We use a piecewise-linear kernel for the aortic test section and a three-point B-spline kernel for the valve leaflets as regularized delta functions (see Supplemental Materials Section D). The time step size starts at $\Delta t = 7.5 \times 10^{-6}$ s, and it is systematically reduced if needed to avoid instabilities related to our time stepping scheme. The penalty parameters $c_{{\text {wall}}}$ and $\kappa _{{\text {wall}}}$ in Eqs. (7) and (8) detailed in “Aortic Test Section” are empirically determined as approximately the largest values allowed by our explicit time stepping algorithm at the time step sizes used in the simulations. Supplemental Materials Section F shows results from a grid convergence study to quantify the level of consistency in our numerical results and to justify the chosen level of grid resolution for the final results reported here.

### Software Infrastructure

FSI simulations use the IBAMR software infrastructure, which is a distributed-memory parallel implementation of the IB method with adaptive mesh refinement (AMR).^27^,^35^ IBAMR uses SAMRAI^33^ for Cartesian grid discretization management, libMesh^41^ for FE discretization management, and PETSc^4^ for linear solver infrastructure.

## Results

We perform corresponding experiments and simulations using a pulse rate of 70 beats per minute in all cases. We use 10 consecutive cycles of experimental pressure and flow rate waveforms for both valves to characterize the reduced-order models that provide driving and loading conditions for the three-dimensional FSI models. The stroke volumes of the average flow waveforms are $69.4 \pm 0.4$ and $71.6 \pm 0.7\,{\text {mL}}$ for the porcine and bovine pericardial BHVs, respectively. We assess the computational results by comparisons to available experimental data, including flow rates, upstream and downstream pressures, and leaflet kinematics. The computational models also provide detailed flow patterns and leaflet stress distributions, which are not readily available in the present experimental models.

**Figure 4:**
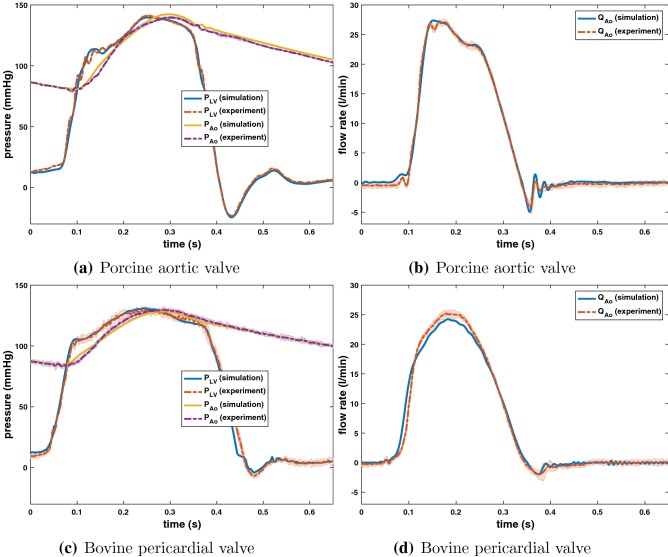
Comparisons between simulated and experimental pressure and flow rate waveforms for the porcine aortic valve (a, b) and bovine pericardial valve (c, d). The experimental waveforms shown are the average waveforms over 10 consecutive cycles of data, with shaded regions showing where 95% of the data fall. The experimental and computational stroke volumes for the porcine aortic valve are $69.4 \pm 0.4$ and $72.7\,{\text {mL}},$ respectively, and $71.6 \pm 0.7$ and $72.1\,{\text {mL}}$ for the bovine pericardial valve. The maximum experimental pressure differences during forward flow for the porcine aortic and bovine pericardial valves are $22.8 \pm 0.2$ and $19.7 \pm 0.5\,{\text {mmHg}},$ respectively. The maximum computational pressure differences during forward flow are $22.4$ and $16.4\,{\text {mmHg}},$ respectively.

Figure 4 shows comparisons between simulated and experimental pressures and flow rates for both valves, which are in good agreement. To quantify this, we calculate the discrepancy between the simulation data and the mean experimental data by

9 $$ \Delta M_{q} = \frac{\left\| M^{{\text {simulation}}} - M^{{\text {experiment}}} \right\| _{L^{q}(0,T)}}{\left\| M^{{\text {experiment}}} \right\| _{L^{q}(0,T)}}, \quad q = 2, \infty , $$

in which (0, *T*) indicates an integral over time and $M^{{\text {experiment}}}$ is the experimental data averaged over 10 cycles. Comparisons are shown in Table 1.

**Table 1:** Comparisons of normalized $L^{2}$- and $L^{\infty }$-norms of discrepancies in the bulk measurements between simulation and experiment.

	Porcine ($L^{2},$ %)	Pericardial ($L^{2},$ %)	Porcine ($L^{\infty },$ %)	Pericardial ($L^{\infty },$ %)
$P_{{\text {LV}}}$	3.4	4.6	8.6	14.4
$P_{{\text {Ao}}}$	1.9	1.4	4.5	3.6
$Q_{{\text {Ao}}}$	4.7	8.6	9.5	20.2

Comparisons of $L^{2}$- and $L^{\infty }$-norms of the discrepancies in the bulk measurements from simulation and experiment shown in Fig. 4, normalized by the $L^{2}$- and $L^{\infty }$-norms of the measurements from the experiment

The computational stroke volumes are 72.7 and 72.1 mL for the porcine and bovine pericardial BHVs, which are 4.68 and 0.71% larger than the mean experimental stroke volumes, respectively. The maximum experimental pressure differences during forward flow for the porcine aortic and bovine pericardial valves are $22.8 \pm 0.2$ and $19.7 \pm 0.5\,{\text {mmHg}},$ respectively. The maximum computational pressure differences during forward flow are $22.4$ and $16.4\,{\text {mmHg}},$ which correspond to differences of 2.0 and 16.5% compared to the experimental pressure difference in the mean pressure waveforms.

**Figure 5:**
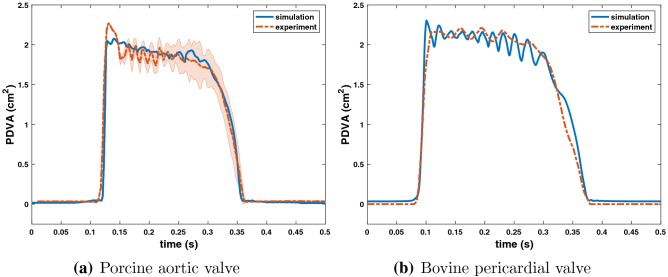
Comparisons between simulated and experimental projected dynamic valve area (PDVA) for the porcine aortic (a) and bovine pericardial (b) valves. (a) The experimental data (acquired using the custom apparatus depicted in Fig. 1b) are manually aligned with the beginning of the valve opening with the simulation data. The experimental PDVA measurement shown is the average PDVA over 10 consecutive cycles of data with shaded region showing where 95% of the data fall. (b) The experimental data are acquired using a high-speed videographic method, from which we reconstruct PDVA data using automatic image analysis using DataTank. Videographic data are available for only a single cycle for the pericardial BHV.

Figure 5 uses PDVA to compare the leaflet kinematics in the experimental and computational models. The experimental data are systematically shifted in time to align with the simulation data at the beginning of valve opening. The timing of opening and closing are in excellent agreement, and the open area is also in excellent agreement. There are some differences in the fluttering frequency, and we discuss potential sources of these discrepancies in the “Discussion” section.

**Figure 6:**
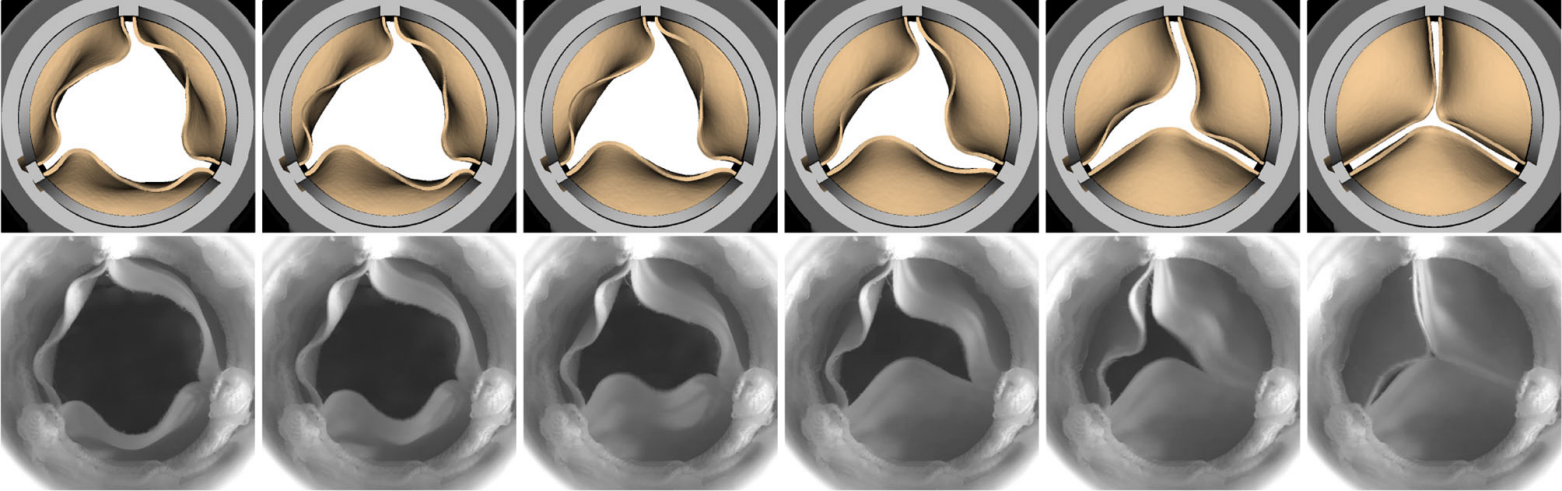
Detailed comparison of the bovine pericardial valve leaflet kinematics during closure in the simulation (top) and experiment (bottom). The simulation captures the behavior of each of the leaflets closing one at a time (order: bottom $\rightarrow$ right $\rightarrow$ left leaflet) as observed in the experiment. The time increment between frames for simulation is 9.6 ms.

Figure 6 compares the leaflet kinematics of the bovine pericardial valve during closure in our simulation to images acquired *via* high-speed videography. We observe that each of the leaflets close one at a time in the experiment as well as in the simulation.

**Figure 7:**
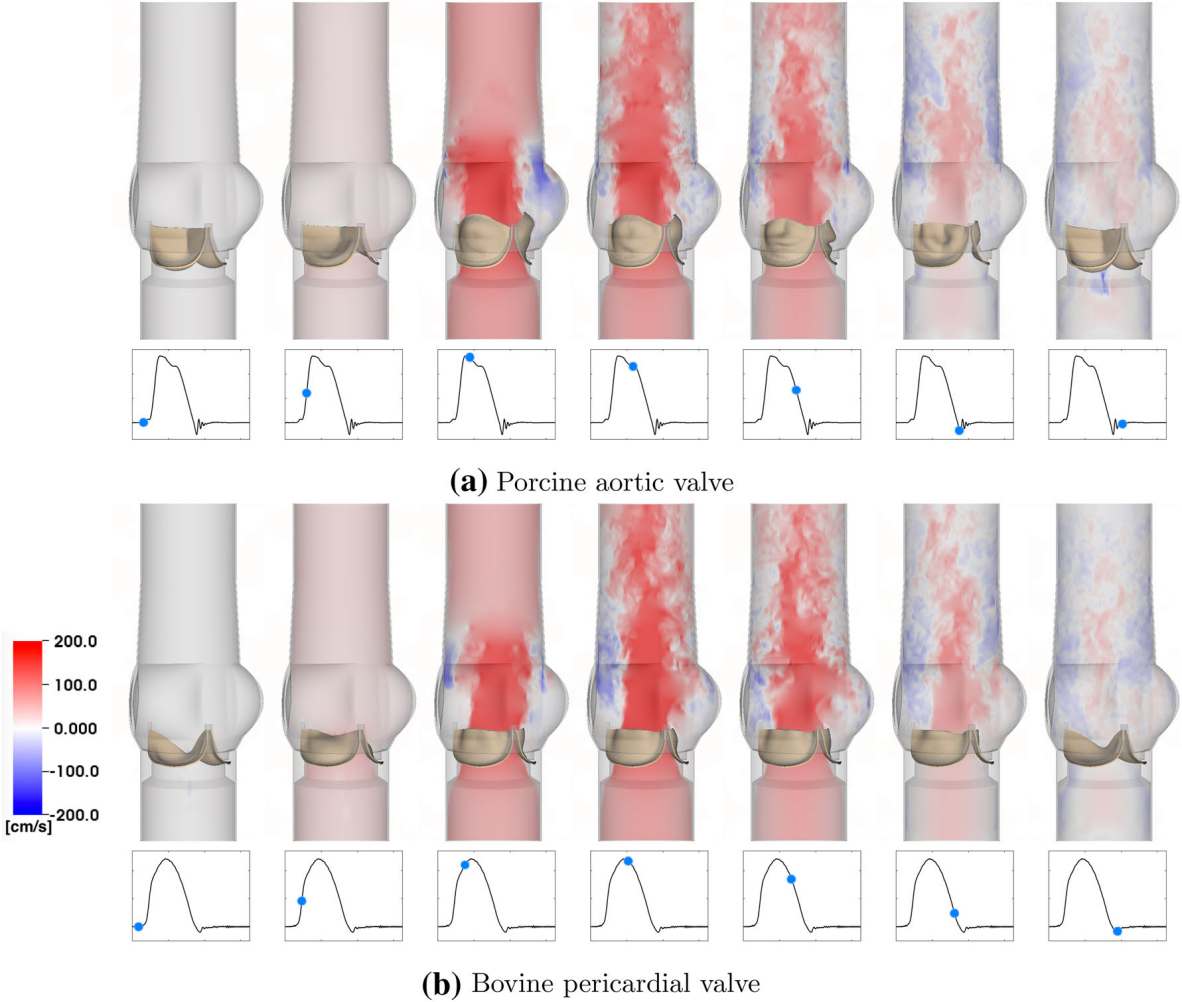
Cross-section view of simulated flow patterns using the porcine aortic (a) and bovine pericardial (b) valve models. The color shows the axial velocity through the aortic test section at the center plane, with red indicating forward flow and blue indicating reverse flow. (a) $Re_{{\text {peak}}} = \text{20,576}.$ (b) $Re_{{\text {peak}}} = \text{19,330}.$ The time increment between frames is 57.6 ms.

Figure 7 provides detailed flow patterns generated in the FSI simulations of the porcine aortic (Fig. 7a) and bovine pericardial (Fig. 7b) BHVs. We observe that the large-scale flow features are similar in both cases. We classify the flow regime of our simulations by computing the peak Reynolds number $Re_{{\text {peak}}},$

10 $$ Re_{{\text {peak}}} = \frac{\rho Q_{{\text {peak}}}D}{\mu A}, $$

in which $\rho$ is the density, $Q_{{\text {peak}}}$ is the peak flow rate, *D* is the diameter of the aortic test section, $\mu$ is the dynamic viscosity of the fluid, and *A* is the cross-sectional area of the aortic test section. $Re_{{\text {peak}}}$ is 20,576 and 19,330 for the porcine tissue and bovine pericardial BHV, respectively.

**Figure 8:**
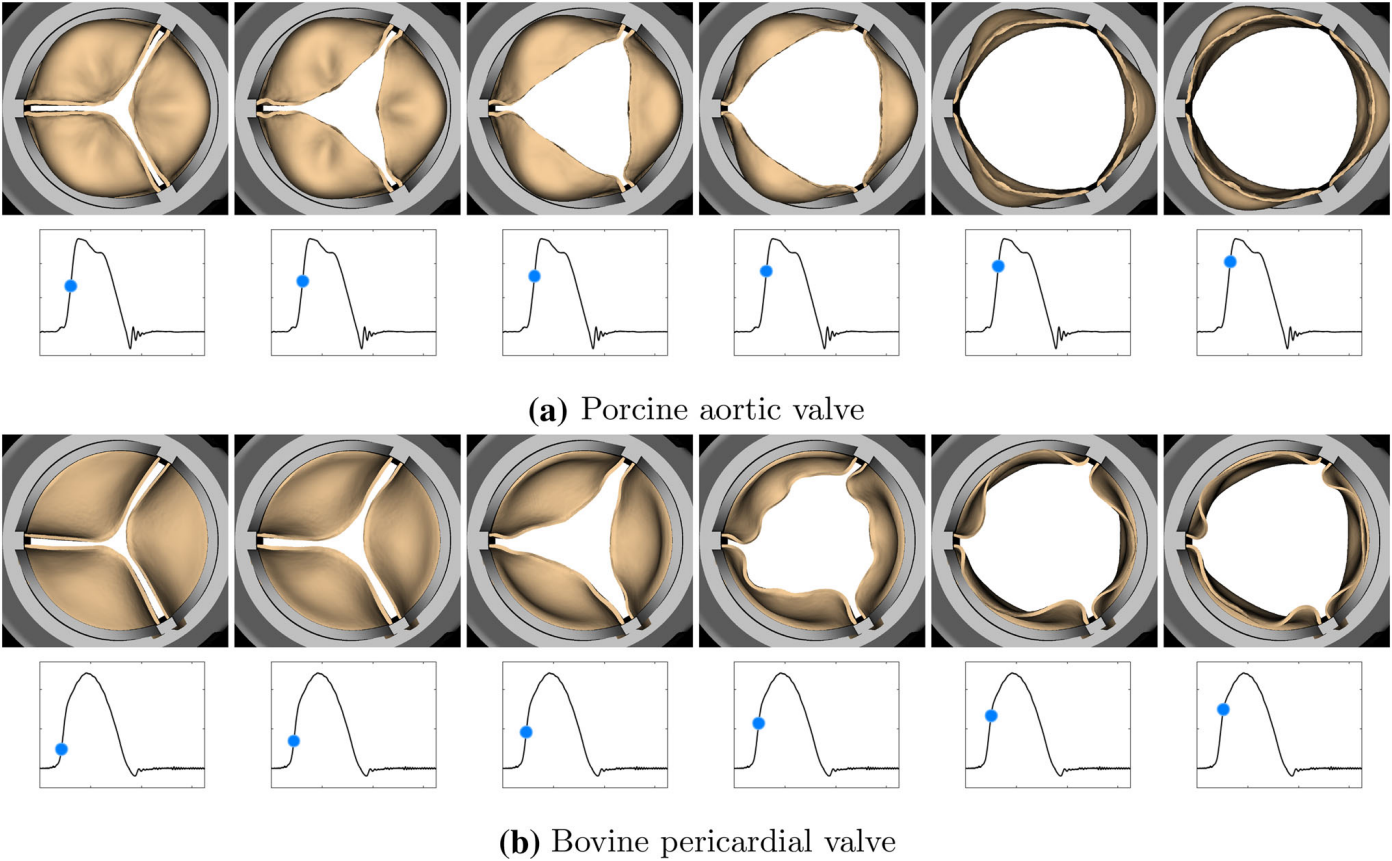
Leaflet kinematics of the porcine aortic (a) and bovine pericardial (b) valves during opening. (a) The time increment between frames is 1.92 ms. (b) The time increment between frames is 3.84 ms.

**Figure 9:**
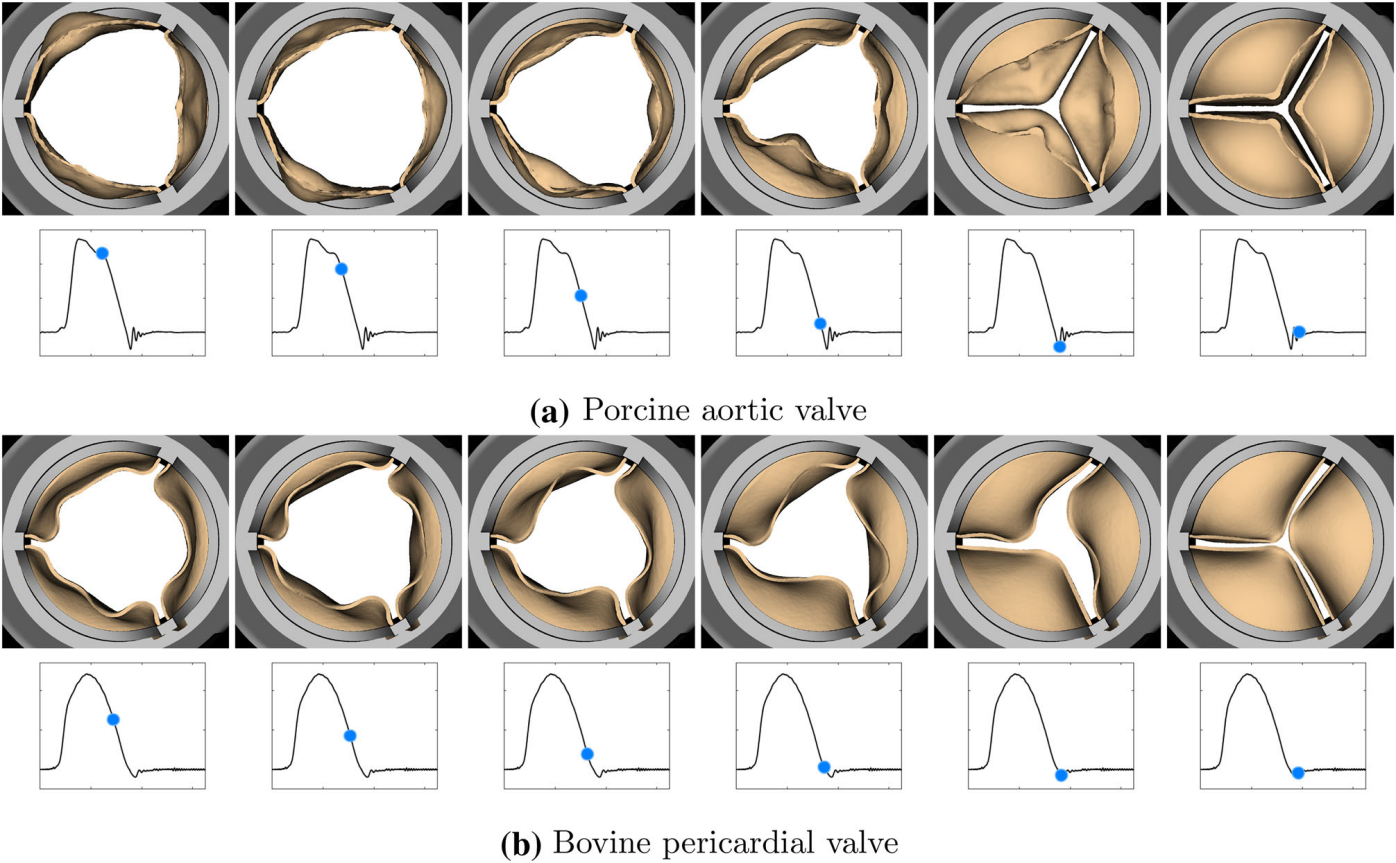
Leaflet kinematics of the porcine aortic (a) and bovine pericardial (b) valves during closure. (a) The time increment between frames is 9.6 ms. (b) The time increment between frames is 19.2 ms.

We also compare the leaflet kinematics of the porcine tissue (Figs. 8a and 9a) and bovine pericardial (Figs. 8b and 9b) valves. One important difference between the two valves is the symmetry breaking in the pericardial leaflets, especially during closure. Because of the asymmetric fiber architecture in each of its leaflets, the bovine pericardial valve shows a swirling motion during closure, as also observed in real pericardial BHVs.^19^

**Figure 10:**
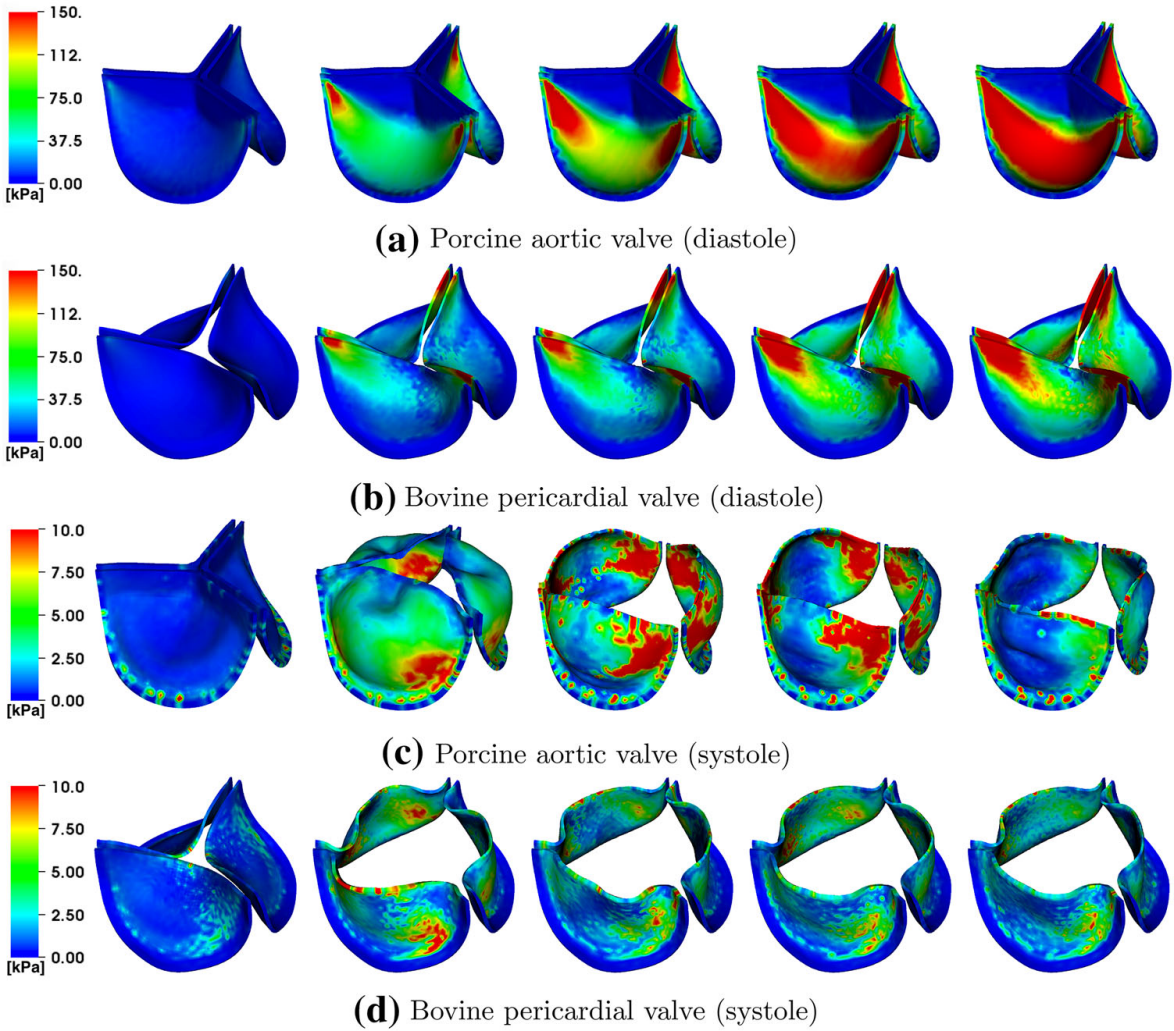
von Mises stress (kPa) on the porcine aortic (a, c) and bovine pericardial (b, d) valves during diastole (a, b) and systole (c, d). The time increment between frames in panels (a, b) is 30.72 ms, and the time increment between frames in panels (c, d) is 11.52 ms.

Differences between the porcine valve and bovine pericardial valve resulting from different fiber architectures are also clear in the stress distributions (Fig. 10). The von Mises stresses on the valves are distributed according to the anisotropic material responses. The porcine valve shows a stress distribution that is aligned from commissure to commissure when the valve is loaded in diastole (Fig. 10a). The stress is also distributed symmetrically on each porcine leaflet during valve opening (Fig. 10c), which is again concentrated around the commissures. In contrast, the bovine pericardial valve shows an asymmetric stress distribution, and the von Mises stresses are concentrated at one of each leaflet’s commissures. The location where stress is the highest on the leaflet occurs where the fibers collect at the commissures (Fig. 10b). The opposite is true during valve opening, when the highest leaflet stresses occur near the free edges of the leaflets (Fig. 10d). These patterns appear because the parts of the leaflets with less commissural support experience larger deformations.

## Discussion

This study has developed FSI models of BHVs in an experimental pulse-duplicator platform. Our leaflet models include anisotropic descriptions of the leaflet biomechanics that are based on experimental tensile test data. In addition, we use experimental pressure and flow data obtained from the pulse-duplicator systems to establish realistic boundary models for the detailed FSI models. These boundary models are calibrated in isolation from the rest of the system, independent of the FSI model of the valves. The simulated pressures, flow rates, and leaflet kinematics all emerge from integrating these three model components, and the motion of the leaflets, including the timing of valve opening and closing, is not prescribed. Further, because the flow rate is not imposed in the model, and because the time-dependent configuration of the valve determines the resistance of the aortic test section, achieving both pressures and flow rates that are in good agreement with the experimental data represents a nontrivial test of the model.

The numerical results detailed in Fig. 4 show excellent agreement in flow rates, pressures, valve open areas, and the timing of valve opening and closure between the simulations and experiments. For instance, the experimental flow rate oscillations that are present during valve closure in the porcine aortic valve (Fig. 4b) are physical because they come from the interaction between the momentum of the fluid and the compliance of the system, and these oscillations are captured by the computational model. We observe from the bovine pericardial BHV case that a more comprehensive reduced-order model of the upstream components of the pulse-duplicator system, including the pump, VIA, left ventricle, left atrium, and mitral valve, allows us to capture system dynamics, including closure (Fig. 4d), more completely. Table 1 shows the $L^{2}$- and $L^{\infty }$-norms of the discrepancies in the simulation results relative to the experimental measurements. In particular, the $L^{2}$-differences demonstrate good quantitative agreement, in which the relative discrepancies are within 4.7% for the porcine aortic valve and 8.6% for the bovine pericardial valve. These results show that our valve models generate leaflet kinematics that yield realistic pressures and flow rates in the fully coupled FSI models.

The experimental and computational leaflet kinematics are assessed using PDVA (Figs. 5a and 5b). The PDVA measurements for the porcine aortic valve indicate reasonable agreement between simulations and experiments. There is a key difference in the techniques used to acquire these data. An electro-optical system (Fig. 1b) was used to measure PDVA directly for the porcine aortic valve. For the bovine pericardial valve, PDVA was calculated indirectly using automatic image analysis *via* DataTank from videographic images. An advantage of the electro-optical subsystem is that it is calibrated with a reference area at the same location as the valve in the test section, but high-speed video is not available for this system. In contrast, although high-speed video is available for the pericardial BHV, as shown in Fig. 6, the image analysis method uses an estimated area-to-pixel scaling. In addition, the frame rate (400 fps) of the high-speed video of bovine pericardial valve is not sufficient to capture the full dynamic response. Consequently, we are missing data needed to resolve the full dynamic waveform. We plan to use higher speed ($\ge 5000\,{\text {fps}}$) videography in future work to quantify leaflet motion more completely. An important limitation of the present model is that we do observe discrepancies between the computational and experimental leaflet fluttering frequencies and amplitudes. BHVs are known to be viscoelastic,^52^ and fully viscoelastic models may be needed to achieve better agreement between simulation and experiment. At present, however, experimentally constrained viscoelastic models of BHV biomaterials suitable for three-dimensional mechanical analyses appear to be lacking. The structural models could be improved by incorporating additional experimental data that characterize the flexural properties of the valve leaflets.

Detailed comparisons of the leaflet kinematics for the bovine pericardial BHV showed that the leaflets closed one at a time both in the experiment and simulation (Fig. 6). This result indicates that the overall kinematics are in reasonable agreement between the experiment and our model. We speculate that this may be because each of the leaflets has slightly different size and geometric features, along with the contribution from its fiber structure. Alternatively, flow instabilities may induce the sequential closure of the valve leaflets.

The large-scale flow features for the porcine aortic and bovine pericardial valves are similar (Fig. 7). We also observe additional small-scale turbulent flow features at current spatial resolutions, which are also present in physiological flow regimes.^23^$Re_{{\text {peak}}}$ for both cases is approximately 20,000,  which clearly motivates the need for further studies on the treatment of turbulence in these types of models. In the present study, we perform implicit large-eddy simulation (ILES) using high-resolution slope limiters, based on the piecewise parabolic method,^14^,^15^,^45^,^57^ to model the flow field. Explicit LES methods have not yet been completely developed for the present IB approach to FSI. We plan to compare ILES^14^,^15^,^45^,^57^ and explicit LES^6^,^59^ models for cardiovascular flows in future work. The similarity in the large-scale flow features for the two BHVs suggests that it is important to consider the leaflet kinematics in addition to the flow patterns in comparing different BHVs. Studying leaflet kinematics could also be important in identifying the factors that affect the durability of different BHVs.

Differences in the porcine and bovine pericardial leaflet kinematics are particularly prominent during leaflet closure (Figs. 8 and 9). Specifically, the twisting motion during closure that we see in the bovine pericardial valve results from the asymmetric alignment of its fibers. This is also evident in stress analyses of the leaflets. Differences in stress distributions (Fig. 10) are caused by the reduced strain at one of two commissure points of each of the bovine pericardial leaflets, whereas the stress distributions in the porcine BHV model reflects the symmetric fiber architecture of the porcine valve cusps. Also, unlike the porcine aortic valve, the pericardial valve lacks nodes of Arantius, which are intrinsic anatomic structures in native aortic valve leaflets. It has been hypothesized that the nodes may play a role in distributing and equalizing leaflet closing stresses.^1^ We also observe that the porcine BHV leaflets experience higher stresses, which may also explain why porcine BHVs have a greater tendency to develop leaflet tears with regurgitation compared to bovine pericardial BHV.^2^,^58^ The stress concentrations at the commissures of the bovine pericardial BHV also agrees with the known failure regions for bovine pericardial BHVs.^44^,^63^

The present study has several limitations. Although we report 10 consecutive cycles of experimental data, one future aim is to better quantify the uncertainty associated with cycle-to-cycle variability in the experiments, and to integrate this uncertainty into our computational models. We shall also quantify variability in the leaflet kinematics along with variability in the pressure and flow rate measurements by collecting high-speed video and data over multiple cycles. We also have not yet systematically performed sensitivity analysis or uncertainty quantification of the model. Performing such analyses is crucial for establishing the credibility of the model but is challenging because of the substantial computational requirements of such analyses. We also plan to validate the flow fields by particle image velocimetry (PIV) under different conditions, as well as using a blood analogue (dynamic viscosity of 3–$4\,{\text {cP}}$) as our test fluid. With PIV, we also plan to perform direct comparison between computational and experimental flows in steady state cases in which we expect to obtain converged mean flows. This will help us quantify the accuracy our model by comparing time-averaged flows as well as turbulence kinetic energies to compare both large- and small-scale flow features to those of the experiments. In this study, we also use homogeneous structural models that omit descriptions of the discrete layers of the valve leaflets, which will affect how the leaflets deform.^10^,^53^ Therefore we shall also further validate specifically the leaflet kinematics by focusing on quasi-static BHV deformations, as done by Sun *et al*.,^66^ or by comparing the leaflet kinematics with reconstructions of leaflet deformations from *in vitro* experiments, as done by Iyengar *et al*.^36^ or Sugimoto *et al*.^65^ Other potential validation tests include assessing BHV performance using additional hydrodynamics performance measures (pressure drop, effective orifice area, *etc*.).^43^,^51^,^55^

In summary, this study describes work to model BHVs in an experimental pulse-duplicator platform that is used in various settings to assess their performance. Ultimately, fully validated FSI models of BHVs may be used to develop a high-fidelity model for predicting dynamic performance amongst different valve designs and addressing persistent challenges posed by current BHV designs. They could also facilitate matching patient requirements with valve performance specifics and the development of regulatory guidelines for evaluation of novel designs. This methodology may also be extended and further validated to be applicable to study the effectiveness of TAVR devices with varying degrees of intra- and paravalvular leak, reduced leaflet mobility, subclinical valve thrombosis, and potential pannus formation.^18^

## Electronic supplementary material

Below is the link to the electronic supplementary material.

Supplementary material 1 (PDF 17863 KB).
