# Supplementary material for: Fluid–Structure Interaction Models of Bioprosthetic Heart Valve Dynamics in an Experimental Pulse Duplicator
Source: Ann Biomed Eng. 2020 Feb 7;48(5):1475–90. doi: 10.1007/s10439-020-02466-4 (PMC7154025; doi:10.1007/s10439-020-02466-4)
Supplement: Supplementary file 1 — Supplementary material 1 (PDF 17863 KB). [file 10439_2020_2466_MOESM1_ESM.pdf]

# Supplemental Materials

## A Reconstruction of the Bovine Pericardial BHV Geometry

The pericardial valve geometry was reconstructed from micro-CT images of a Carpentier-Edwards PERIMOUNT RSR Model 2800 surgical aortic heart valve. The DICOM files, consisting of approximately 500 slices of the 25 mm valve after micro-CT scanning (Scanco Medical; scan parameters: 90 kV; 88 $\mu$A; voxel size 24.6 $\mu$m), were imported into Mimics (Materialise Inc., Leuven, Belgium) for further segmentation and reconstruction. Segmentation and reconstruction operations in Mimics involved first thresholding to remove the dense metal and its artifacts from all the slices, and then cavity filling and region growing to reconstruct the continuity in the leaflet material. These operations helped reconstruct the three-dimensional shape of the leaflets. Other segmentation morphology operations were used to ensure that the leaflet thickness was consistent with the measured thickness of the leaflet. After these operations, the leaflet masks were smoothed, wrapped, and smoothed again prior to importing into Materialise 3-matic (Materialise Inc., Leuven, Belgium). Further processing using design tools in Materialise 3-matic was necessary to sketch and sweep edges of the leaflet to create a smooth outline of the entire leaflet.

## B Parameter Fitting for the Valve Material Models

We perform model fits for the constitutive model of the valves detailed in the "Leaflet Mechanics" section in the main text using experimental data from Billiar and Sacks [1,2] for the porcine tissue BHV and from Kim et al. [3] for the bovine pericardial BHV. In the biaxial tests of Billiar and Sacks, the specimens were mounted onto the testing device so that the radial and circumferential directions were aligned with the direction of the applied forces, as shown in Figure S1a. Kim et al. used a stress-control biaxial testing method on a gluteraldehyde-treated bovine pericardial tissue sample. As shown in Figure S1c, the specimen was aligned at 45° to the direction of the applied forces, mimicking the fiber alignment of the pericardial valve leaflets. To determine constitutive model parameters, we assume that the solid is incompressible, compute the second Piola-Kirchhoff stress ($\mathbb{S}$), and compare it to the experimental values for given values of the Green-Lagrange strain $\mathbb{E} = \frac{1}{2}(\mathbb{C} - \mathbb{I})$. The stress is computed by

$$\mathbb{S} = \mathbb{S}^{\text{dev}} - p\mathbb{C}^{-1}, \tag{S1}$$

$$\mathbb{S}^{\text{dev}} = 2\frac{\partial W}{\partial \mathbb{C}}, \tag{S2}$$

$$p = \frac{\mathbb{S}_{33}^{\text{dev}}}{(\mathbb{C}^{-1})_{33}}, \tag{S3}$$

in which $W = W_{\text{iso}} + W_{\text{aniso}}$ is the Holzapfel-Gasser-Ogden model described by (4) and (5) in the "Leaflet Mechanics" section of the main text, $\mathbb{S}^{\text{dev}}$ is the deviatoric part of the second Piola-Kirchhoff stress, $\mathbb{C} = \mathbb{F}^T\mathbb{F}$ is the Cauchy-Green strain, $\mathbb{F}$ is the deformation gradient tensor, and $p$ is the pressure assuming that the specimen is acted on only by in-plane loads. We use lsqcurvefit in MATLAB (The MathWorks, Inc., Natick, MA, USA), a nonlinear least-squares solver, to determine the model parameters. Figures S1b and S1d show constitutive model fits obtained using this approach.

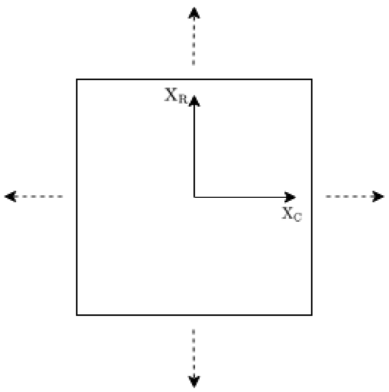


**(a)**

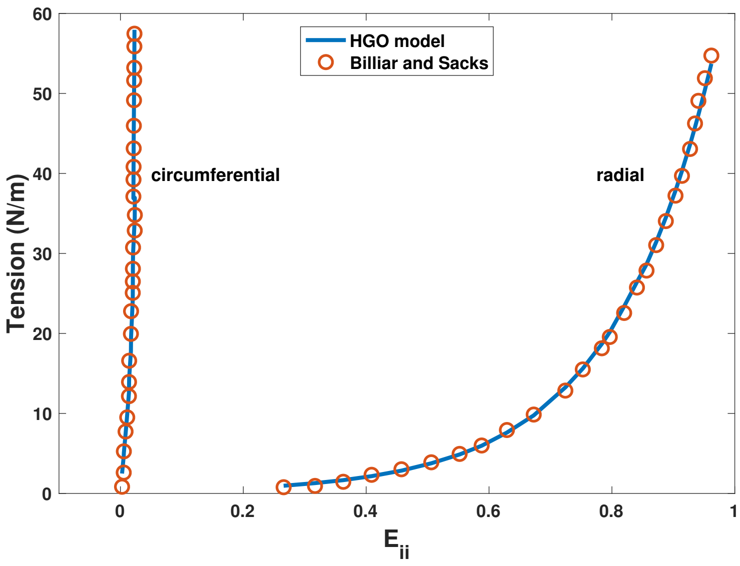


**(b)**

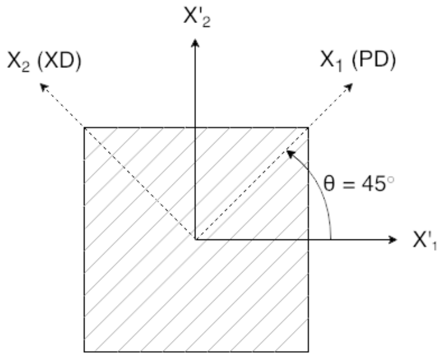


**(c)**

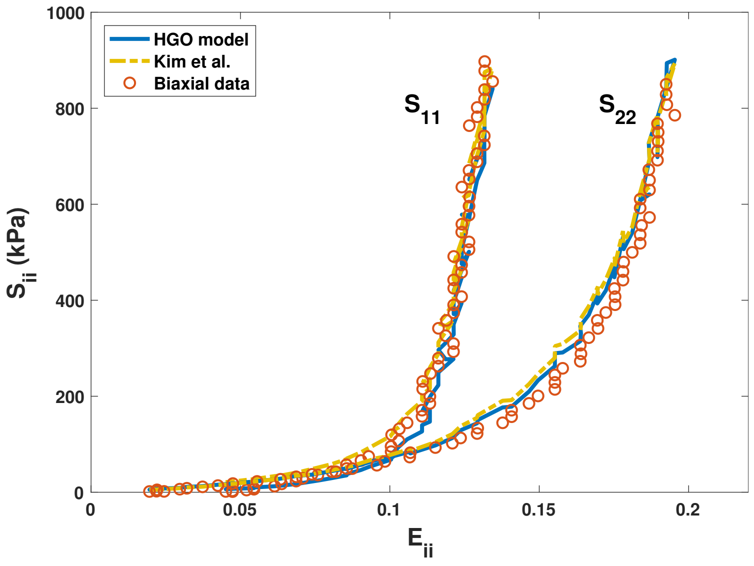


**(d)**

**Figure S1:** (a) Schematic of the biaxial tensile test of Billiar and Sacks [1,2] for porcine aortic valve tissue specimens to study their material response. $X_C$ is the circumferential axis, and $X_R$ is the radial axis. The dashed lines are the directions in which forces were applied. (b) Parameter fitting for the porcine aortic valve using the experimental tensile test data from Billiar and Sacks [1,2] for glutaraldehyde-fixed porcine aortic valves. (c) Schematic of the biaxial tensile tests of Kim et al. [3] for bovine pericardium tissue specimens to study their material response. $X_1$ is the preferred mean fiber direction (PD) also shown by gray lines, $X_2$ is the cross-preferred fiber direction (XD), and $X'_1$ and $X'_2$ are the directions in which forces were applied. (d) Parameter fitting for the bovine pericardial valve using the equibiaxial data from Kim et al. [3] compared to the plot using parameters determined by Kim et al. using a finite element model of the biaxial test.

# C Parameter Fitting for the Windkessel Model

Pulse-duplicator system components upstream of the aortic test section, including the resistance and compliance of the pump, the viscoelastic impedance adapter (VIA) subsystem, and the left ventricular chamber of the pulse duplicator, are described by a three-element Windkessel (R-C-R) model for the porcine aortic valve, whereas a more detailed model is used for the bovine pericardial valve because additional experimental data are available for the bovine pericardial BHV cases. See Figure 3 of the main text. We remark that the VIA subsystem includes tunable compliance chambers (see Figure 1b of the main text) that help to ameliorate unphysiological pressure oscillations in the left ventricular pressure [4]. The upstream model used in the porcine aortic valve simulations is

$$C_{\mathrm{VIA}} \frac{\mathrm{d}P_{\mathrm{VIA}}}{\mathrm{d}t} = \frac{P_{\mathrm{pump}} - P_{\mathrm{VIA}}}{R_1} + Q_{\mathrm{LV}}, \tag{S4}$$

$$\frac{P_{\mathrm{LV}}}{R_2} = \frac{P_{\mathrm{VIA}}}{R_2} + Q_{\mathrm{LV}}, \tag{S5}$$

in which $C_{\mathrm{VIA}}$, $R_1$, and $R_2$ characterize the VIA system, and $Q_{\mathrm{LV}}$ and $P_{\mathrm{LV}}$ are the volumetric flow rate and pressure, respectively, at the inlet of the aortic test section model. $P_{\mathrm{pump}}$ is derived from (S4) and (S5) using experimental measurements of $Q_{\mathrm{LV}}$ and $P_{\mathrm{LV}}$. The upstream model used in the bovine pericardial valve simulations is

$$C_{\mathrm{VIA}_1} \frac{\mathrm{d}P_{\mathrm{VIA}_1}}{\mathrm{d}t} = Q_{\mathrm{pump}} - \frac{P_{\mathrm{VIA}_1} - P_{\mathrm{VIA}_2}}{R_{\mathrm{VIA}}}, \tag{S6}$$

$$C_{\mathrm{VIA}_2} \frac{\mathrm{d}P_{\mathrm{VIA}_2}}{\mathrm{d}t} = \frac{P_{\mathrm{VIA}_1} - P_{\mathrm{VIA}_2}}{R_{\mathrm{VIA}}} - \frac{P_{\mathrm{VIA}_2} - P_{\mathrm{LA}} + Q_{\mathrm{LV}} R_{\mathrm{MV}}}{R_{\mathrm{out}} + R_{\mathrm{MV}}}, \tag{S7}$$

$$P_{\mathrm{LV}} = \frac{P_{\mathrm{VIA}_2} R_{\mathrm{MV}} + P_{\mathrm{LA}} R_{\mathrm{out}} - Q_{\mathrm{LV}} R_{\mathrm{out}} R_{\mathrm{MV}}}{R_{\mathrm{out}} + R_{\mathrm{MV}}}, \tag{S8}$$

in which $C_{\mathrm{VIA}_1}$, $C_{\mathrm{VIA}_2}$, $R_{\mathrm{VIA}}$, and $R_{\mathrm{out}}$ characterize the VIA system, $R_{\mathrm{MV}}$ characterizes the resistance at the mitral position, $P_{\mathrm{LA}}$ is the left atrial pressure, $Q_{\mathrm{pump}}$ is the prescribed volumetric flow rate of the pump, and $Q_{\mathrm{LV}}$ and $P_{\mathrm{LV}}$ are the volumetric flow rate and pressure, respectively, at the inlet of the aortic test section model.

The downstream loading conditions of the pulse duplicator are also described by a three-element Windkessel model,

$$C \frac{\mathrm{d}P_{\mathrm{Wk}}}{\mathrm{d}t} = Q_{\mathrm{Ao}} - \frac{P_{\mathrm{Wk}}}{R_{\mathrm{p}}}, \tag{S9}$$

$$P_{\mathrm{Ao}} = P_{\mathrm{Wk}} + Q_{\mathrm{Ao}} R_{\mathrm{c}}, \tag{S10}$$

in which $C$ is the compliance, $R_{\mathrm{c}}$ is the characteristic resistance, $R_{\mathrm{p}}$ is the peripheral resistance, $P_{\mathrm{Wk}}$ is the Windkessel pressure, and $Q_{\mathrm{Ao}}$ and $P_{\mathrm{Ao}}$ are the volumetric flow rate and pressure, respectively, at the outlet of the test section. The Windkessel parameters $R_{\mathrm{c}}$, $R_{\mathrm{p}}$, and $C$ are calibrated using experimental measurements of $Q_{\mathrm{Ao}}$ and $P_{\mathrm{Ao}}$. We use the nonlinear optimization tool fmincon in MATLAB to determine the model parameters by comparing $P_{\mathrm{Ao}}$ from (S9) and (S10) with that of experimental system (Figure S2). When calibrating the reduced-order models, the experimental measurements of $Q_{\mathrm{Ao}}$ are used as inputs to the Windkessel models.

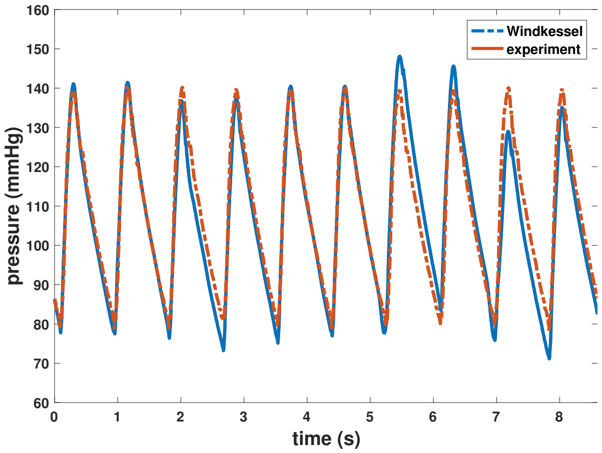


**(a)** Porcine aortic valve (downstream)

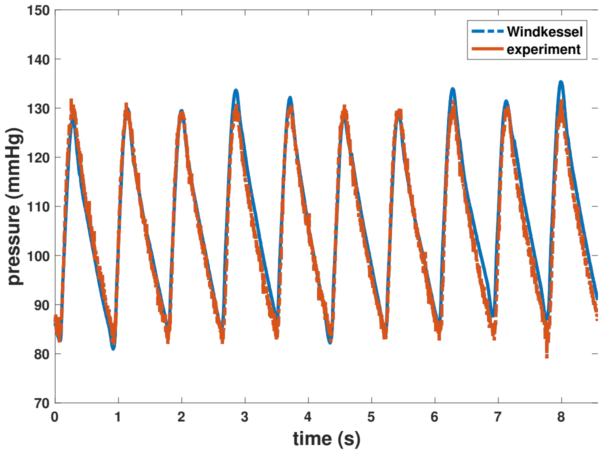


**(b)** Bovine pericardial valve (downstream)

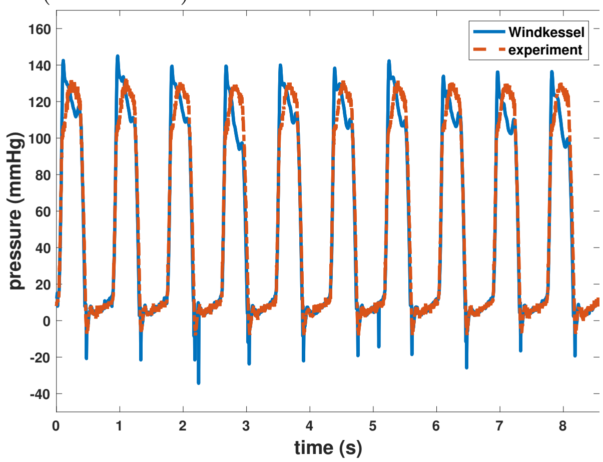


**(c)** Bovine pericardial valve (upstream)

**Figure S2:** (a)–(b) The three-element Windkessel model fits of the experimental downstream pressure (pressure at the outlet) data for both porcine aortic valve and bovine pericardial valve models (Figures 3a and 3b). These fits are obtained using a nonlinear optimization tool fmincon in MATLAB by comparing experimental values of $P_{\mathrm{Ao}}$ to model results obtained by solving (S9) and (S10) with measured values of $Q_{\mathrm{Ao}}$ as inputs to the Windkessel models. (c) Model fit of the experimental upstream pressure (pressure at the inlet) data for the bovine pericardial valve model using a detailed upstream model shown in Figure 3b. These fits are obtained by comparing experimental values of $P_{\mathrm{LV}}$ to model results obtained by solving (S6), (S7), and (S8) with measured values of $Q_{\mathrm{pump}}$, $Q_{\mathrm{LV}}$, and $P_{\mathrm{LA}}$.

A first-order (Godunov) time step splitting is used to couple the three-dimensional FSI model to the reduced-order models, as described by Griffith et al. [5]. At the outlet of the aortic test section, for example, the mean flow rate through the outlet surface computed by the three-dimensional FSI model is provided as an input to the Windkessel model. The pressure generated in the Windkessel model is used, along with the flow rate, to determine the pressure along the outlet surface, which is used as a boundary condition for the three-dimensional model.

# D Immersed Boundary Formulation

## D.1 Continuum Equations

The IB formulation uses an Eulerian description of the momentum, viscosity, and incompressibility of the coupled fluid-structure system, and it uses a Lagrangian description of the deformations, stresses, and resul-

tant forces of the immersed structure. In particular, we model the immersed structure as a viscoelastic solid, in which the viscous stresses in the solid are small compared to elastic stresses, as in previous work by us and others [6–11]. The coupling between Eulerian and Lagrangian variables is mediated by integral transforms with delta function kernels. In the continuous IB formulation, we consider a fixed three-dimensional Eulerian computational domain $\Omega$ that is divided into the time-dependent solid ($\Omega_t^{\mathrm{s}}$) and fluid ($\Omega_t^{\mathrm{f}}$) subdomains, so that $\Omega = \Omega_t^{\mathrm{s}} \cup \Omega_t^{\mathrm{f}}$. Here, $\boldsymbol{x} = (x_1, x_2, x_3) \in \Omega$ are physical coordinates, $\boldsymbol{X} = (X_1, X_2, X_3) \in \Omega_0^{\mathrm{s}}$ are reference coordinates attached to the structure, $\boldsymbol{N}(\boldsymbol{X}) \in \partial\Omega_0^{\mathrm{s}}$ is the outward unit normal to the reference configuration of the solid region at material point $\boldsymbol{X}$, and $\boldsymbol{\chi}(\boldsymbol{X}, t) \in \Omega_t^{\mathrm{s}}$ is the physical position of material point $\boldsymbol{X}$ at time $t$. $\rho$ and $\mu$ are the mass density and viscosity, $\boldsymbol{u}(\boldsymbol{x}, t)$ and $p(\boldsymbol{x}, t)$ are the Eulerian velocity and pressure fields, $\boldsymbol{f}(\boldsymbol{x}, t)$ is the Eulerian elastic force density, $\mathbb{P}(\boldsymbol{X}, t)$ is the first Piola-Kirchhoff elastic stress of the immersed structure, and $\delta(\boldsymbol{x}) = \prod_{i=1}^{3} \delta(x_i)$ is the three-dimensional Dirac delta function. The IB form of the equations of motion for the fluid-structure system is:

$$\rho \frac{\mathrm{D}\boldsymbol{u}}{\mathrm{D}t}(\boldsymbol{x}, t) = -\nabla p(\boldsymbol{x}, t) + \mu \nabla^2 \boldsymbol{u}(\boldsymbol{x}, t) + \boldsymbol{f}(\boldsymbol{x}, t), \tag{S11}$$

$$\nabla \cdot \boldsymbol{u}(\boldsymbol{x}, t) = 0, \tag{S12}$$

$$\begin{aligned} \boldsymbol{f}(\boldsymbol{x}, t) = &\int_{\Omega_0^s} \nabla_{\boldsymbol{X}} \cdot \mathbb{P}(\boldsymbol{X}, t)\, \delta(\boldsymbol{x} - \boldsymbol{\chi}(\boldsymbol{X}, t))\, \mathrm{d}\boldsymbol{X} \\ &- \int_{\partial\Omega_0^s} \mathbb{P}(\boldsymbol{X}, t)\, \boldsymbol{N}(\boldsymbol{X})\, \delta(\boldsymbol{x} - \boldsymbol{\chi}(\boldsymbol{X}, t))\, \mathrm{d}A, \end{aligned} \tag{S13}$$

$$\frac{\partial \boldsymbol{\chi}}{\partial t}(\boldsymbol{X}, t) = \int_{\Omega} \boldsymbol{u}(\boldsymbol{x}, t)\, \delta(\boldsymbol{x} - \boldsymbol{\chi}(\boldsymbol{X}, t))\, \mathrm{d}\boldsymbol{x} = \boldsymbol{u}(\boldsymbol{\chi}(\boldsymbol{X}, t), t), \tag{S14}$$

in which $\frac{\mathrm{D}}{\mathrm{D}t} = \frac{\partial}{\partial t} + \boldsymbol{u} \cdot \nabla$ is the material derivative. Notice that because $\partial \boldsymbol{\chi}/\partial t(\boldsymbol{X}, t) = \boldsymbol{u}(\boldsymbol{\chi}(\boldsymbol{X}, t), t)$ and $\nabla \cdot \boldsymbol{u}(\boldsymbol{x}, t) = 0$, the immersed structure is described in the continuous IB framework as an exactly incompressible material.

## D.2 Numerical Approximations

The computational domain $\Omega$, which includes both the solid and fluid subregions, is described using a block-structured locally refined Cartesian grid consisting of multiple nested levels of Cartesian grid patches. High spatial resolution is used dynamically near fluid-structure interfaces (Figure S3) and near flow features, such as vortices shed from the valve leaflets, that are identified by feature detection criteria that select regions of high vorticity for enhanced spatial resolution. A staggered-grid discretization is used for the incompressible Navier-Stokes that includes a version [12, 13] of the xsPPM7 variant [14] of the piecewise parabolic method (PPM) [15] to approximate the convective term.

In our simulations, we use a regularized delta function $\delta_h(\boldsymbol{x})$ to approximate the singular Dirac delta function $\delta(\boldsymbol{x})$. The three-dimensional regularized delta function is the tensor product of one-dimensional delta functions, $\delta_h(\boldsymbol{x}) = \prod_{i=1}^{3} \delta_h(x_i)$, and the one-dimensional delta function is defined in terms of a basic kernel function via $\delta_h(x) = \frac{1}{h}\varphi\left(\frac{x}{h}\right)$. In our computations, different regularized delta functions are used for the rigid aortic test section and flexible valve leaflets. These choices are based on preliminary benchmarking studies extending the tests reported by Griffith and Luo [11]. For the aortic test section, we use a simple piecewise-linear (PWL) kernel that takes the form,

$$\varphi^{\mathrm{PWL}}(r) = \begin{cases} 1 - r, & 0 \leq r < 1, \\ 0, & 1 \leq r, \end{cases} \tag{S15}$$

and we use a three-point B-spline (BS3) kernel for the valve leaflets,

$$\varphi^{\mathrm{BS3}}(r) = \begin{cases} \frac{3}{4} - r^2, & 0 \leq r < 0.5, \\ \frac{9}{8} - \frac{3}{2}r + \frac{1}{2}r^2, & 0.5 \leq r \leq 1.5, \\ 0, & 1.5 \leq r. \end{cases} \tag{S16}$$

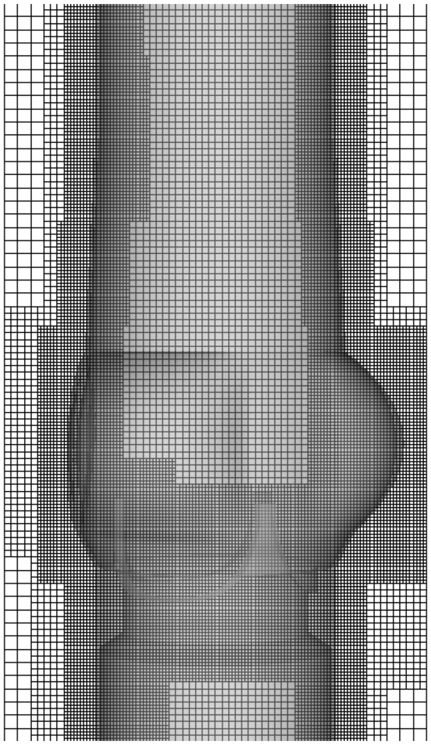

**Figure S3:** The aortic test section in the computational domain. The computational domain is a block-structured adaptively refined Cartesian grid. A snapshot of the locally refined grid is shown on a plane that bisects the aortic test section.

Notice that the gap between the valve leaflets during closure reflects the size of regularized delta function, and in our simulations, the valve is effectively closed during the diastolic phase of the simulated cardiac cycle. This is also shown by Figures 4b and 4d in the main text, in which there is no leakage once the valve is closed. An advantage of the IB formulation of such problems is that it does not require an explicit contact model to simulate contact between the valve leaflets.

# E Stabilization Method for the Hyperelastic Immersed Boundary Method

In the continuum equations of the IB framework (Supplemental Materials Section D.1), the Cauchy stress on the full domain can be seen to take the form

$$\sigma(\boldsymbol{x},t) = \sigma^{\mathrm{f}} + \begin{cases} \mathbb{0}, & \boldsymbol{x} \in \Omega_t^{\mathrm{f}}, \\ \sigma^{\mathrm{e}}, & \boldsymbol{x} \in \Omega_t^{\mathrm{s}}, \end{cases} \tag{S17}$$

which can also be written as

$$\sigma(\boldsymbol{x},t) = \mathrm{dev}[\sigma^{\mathrm{v}}] - p\mathbb{I} + \begin{cases} \mathbb{0}, & \boldsymbol{x} \in \Omega_t^{\mathrm{f}}, \\ \sigma^{\mathrm{e}}, & \boldsymbol{x} \in \Omega_t^{\mathrm{s}}, \end{cases} \tag{S18}$$

in which $\sigma^{\mathrm{e}} = \frac{1}{J}\mathbb{P}\mathbb{F}^T$, $\sigma^{\mathrm{v}} = \mu(\nabla \boldsymbol{u} + \nabla \boldsymbol{u}^T)$ is the viscous stress, the pressure $p$ is a Lagrange multiplier that imposes the incompressibility constraint for both the fluid and structure, and $\mathrm{dev}[\sigma^{\mathrm{v}}]$ is the deviatoric part of $\sigma^{\mathrm{v}}$ that is defined as

$$\mathrm{dev}[\sigma^{\mathrm{v}}] = \sigma^{\mathrm{v}} - \frac{\mathrm{tr}(\sigma^{\mathrm{v}})}{3}\mathbb{I}. \tag{S19}$$

Although the continuous IB equations model the structure as exactly incompressible, the present numerical method is not guaranteed to preserve the divergence of the Eulerian velocity field when that field is evaluated

on the Lagrangian finite element mesh. Consequently, the method only approximately accounts for the incompressibility of the immersed structure, although the accuracy of this approximation improves under grid refinement. Vadala-Roth et al. [16] introduced a volumetric energy in the solid region to stabilize and improve the accuracy of the numerical scheme substantially,

$$\mathbb{\sigma}(\boldsymbol{x},t) = \mathrm{dev}[\mathbb{\sigma}^{\mathrm{v}}] - p\mathbb{I} + \begin{cases} \mathbb{0}, & \boldsymbol{x} \in \Omega_t^{\mathrm{f}}, \\ \mathrm{dev}[\mathbb{\sigma}^{\mathrm{e}}] - p_{\mathrm{stab}}\mathbb{I}, & \boldsymbol{x} \in \Omega_t^{\mathrm{s}}, \end{cases} \tag{S20}$$

in which the stabilization term $p_{\mathrm{stab}} = -\partial U(J)/\partial J$ acts as a pressure that reinforces incompressibility in the solid region in the numerical method. We refer to this as a stabilization method because its effect vanishes under grid refinement. Specifically, in the continuum limit, the structure is automatically incompressible in the IB framework, and so $J \equiv 1$ and $p_{\mathrm{stab}} \equiv 0$. This stabilization method has been tested using standard quasi-static solid mechanics benchmark problems. Sections E.1 and E.2 show two benchmark examples from Vadala-Roth et al. [16] that demonstrate the effect of this stabilization method. These results are compared to and show excellent agreement with the results from a stabilized incompressible finite element solid mechanics formulation. Additional details are provided by Vadala-Roth et al. [16].

## E.1 Compression Benchmark

This benchmark involves a rectangular block with a downward traction of 200 dyn cm$^{-2}$ applied at the center of the top side along with zero horizontal displacement. Zero vertical displacement is imposed at the bottom side of the block, and zero traction is applied at all sides other than the top side [17], as shown in Figure S4a. The block is a neo-Hookean material,

$$\Psi = \frac{c_1}{2}(\bar{I}_1 - 3) + \frac{\kappa_{\mathrm{stab}}}{2}(\ln J)^2, \tag{S21}$$

in which $\bar{I}_1 = \mathrm{tr}(\overline{\mathbb{C}})$ is the first invariant of the modified right Cauchy-Green strain tensor $\overline{\mathbb{C}} = \overline{\mathbb{F}}^T\overline{\mathbb{F}} = J^{-2/3}\mathbb{F}^T\mathbb{F}$. Here, $c_1$ is the shear modulus, and $\kappa_{\mathrm{stab}}$ is the numerical bulk modulus controlled by the shear modulus and the numerical Poisson ratio, $\nu_{\mathrm{stab}}$, via

$$\kappa_{\mathrm{stab}} = \frac{2\mu_{\mathrm{s}}(1+\nu_{\mathrm{stab}})}{3(1-2\nu_{\mathrm{stab}})}, \tag{S22}$$

in which $\mu_{\mathrm{s}}$ is the linearized ($\mathbb{F} = \mathbb{I}$) shear modulus. For the neo-Hookean model, $\mu_{\mathrm{s}} = c_1$. This test uses $c_1 =$ 80.194 dyn cm$^{-2}$ and $\nu_{\mathrm{stab}} = 0.4$. The density and viscosity are $\rho = 1.0$ g cm$^{-3}$ and $\mu = 0.01$ dyn s cm$^{-2}$, respectively. We again emphasize that the numerical bulk modulus, $\kappa_{\mathrm{stab}}$, and the numerical Poisson ratio, $\nu_{\mathrm{stab}}$, act as stabilization parameters, and not as physical parameters of the model, and that their effects vanish under grid refinement. This test shows that using the stabilization method yields superior volume conservation and smooth deformations for the mesh elements (Figures S4b and S4d).

## E.2 Torsion Benchmark

This benchmark involves applying torsion to an elastic beam [18] as shown in Figure S5a. Torsion is applied to the top face of the beam while zero displacement is imposed at the opposite face. Zero traction boundary conditions were used on all the other faces. The elastic beam is a Mooney-Rivlin material,

$$\Psi = c_1(\bar{I}_1 - 3) + c_2(\bar{I}_2 - 3) + \frac{\kappa_{\mathrm{stab}}}{2}(\ln J)^2, \tag{S23}$$

in which $\bar{I}_2 = (\bar{I}_1^2 - \mathrm{tr}(\overline{\mathbb{C}}^2))/2$ is the second invariant of $\overline{\mathbb{C}}$. For the Mooney-Rivlin model, $\mu_{\mathrm{s}} = 2(c_1 + c_2)$. This test uses material parameters $c_1 = 9000$ dyn cm$^{-2}$, $c_2 = 9000$ dyn cm$^{-2}$, and $\nu_{\mathrm{stab}} = 0.4$. The density and viscosity are $\rho = 1.0$ g cm$^{-3}$ and $\mu = 0.04$ dyn s cm$^{-2}$, respectively. This test also shows that the stabilization method gives reasonable deformation (Figure S5c) as well as yielding superior volume conservation (Figures S5b and S5d).

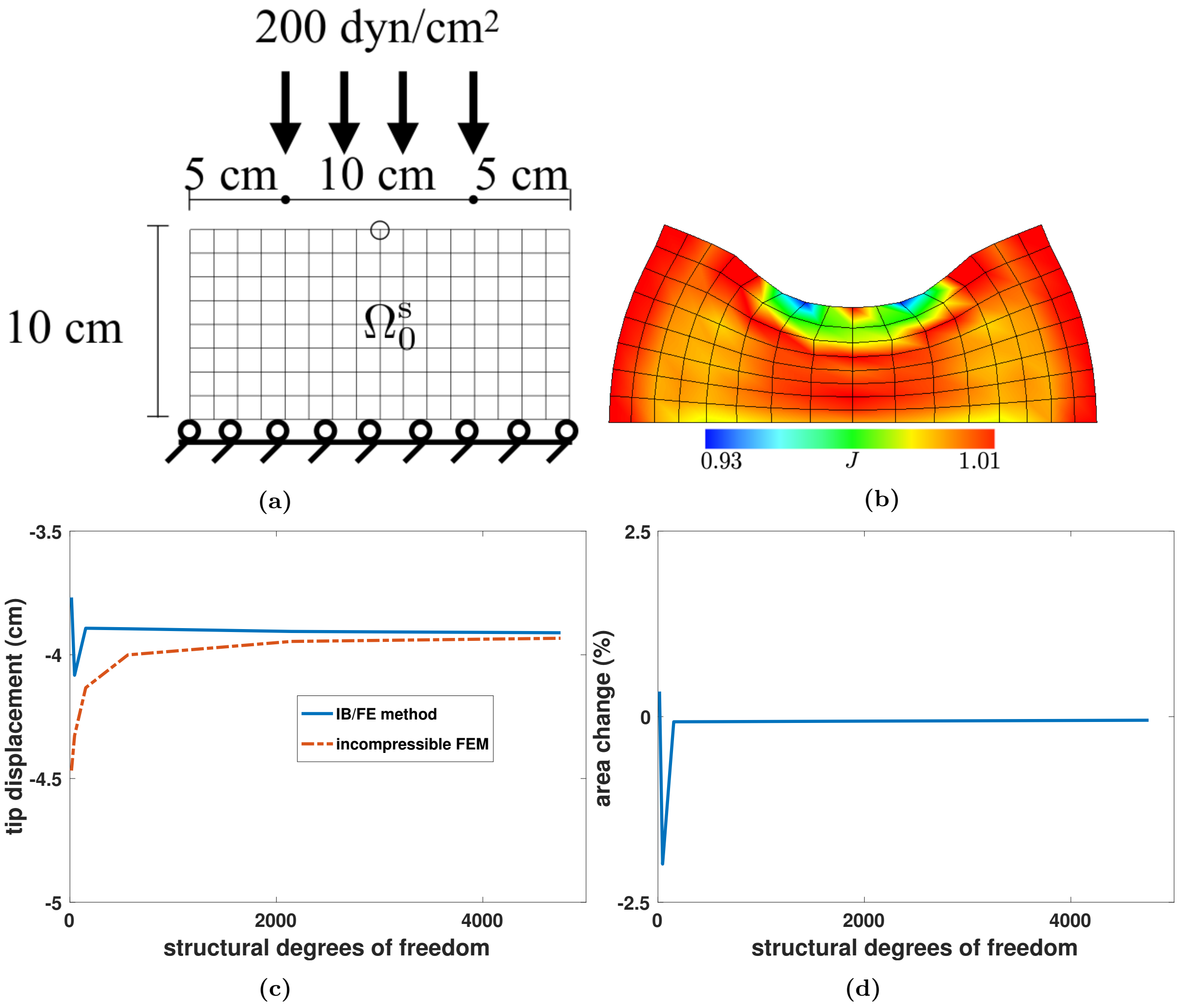


**Figure S4:** (a) Schematic of the compressed block benchmark from Vadala-Roth et al. [16]. A downward traction applied at the center of the top side along with zero horizontal displacement. Zero vertical displacement is imposed at the bottom side of the block, and zero traction is applied at all sides other than the top side, as used by Reese et al. [17]. (b) Deformation of the compressed block for a neo-Hookean material model. The stabilization method results in smooth element deformation as well as excellent volume (area) conservation. The color shows the magnitude of $J = \det(\mathbb{F})$. (c) Displacement of the circled location in panel a compared to result from using standard incompressible structural mechanics formulation. (d) Percent change in total area; see also panel (b).

# F Solution Verification

We perform grid convergence studies to verify the level of consistency in our numerical results. We consider both dynamic and steady flow conditions at three different spatial resolutions. For the dynamic cases, we consider the consistency under grid refinement of all of the measurements reported in the manuscript. For the steady flow conditions, we observe the time-averaged flow rates and the turbulence kinetic energy (TKE) between different resolutions, in addition to bulk measurements.

We use locally refined Cartesian grids with an effective fine-grid resolution that corresponds to a uniform $N \times N \times N$ grid, and we compare results obtained for $N = 192$, 256, and 320. The effective fine-grid Cartesian resolutions are approximately 0.53 mm ($N = 192$), 0.39 mm ($N = 256$), and 0.32 mm ($N = 320$). The average grid-spacings of meshes are 0.5 mm ($N = 192$), 0.4 mm ($N = 256$), and 0.3 mm ($N = 320$) for

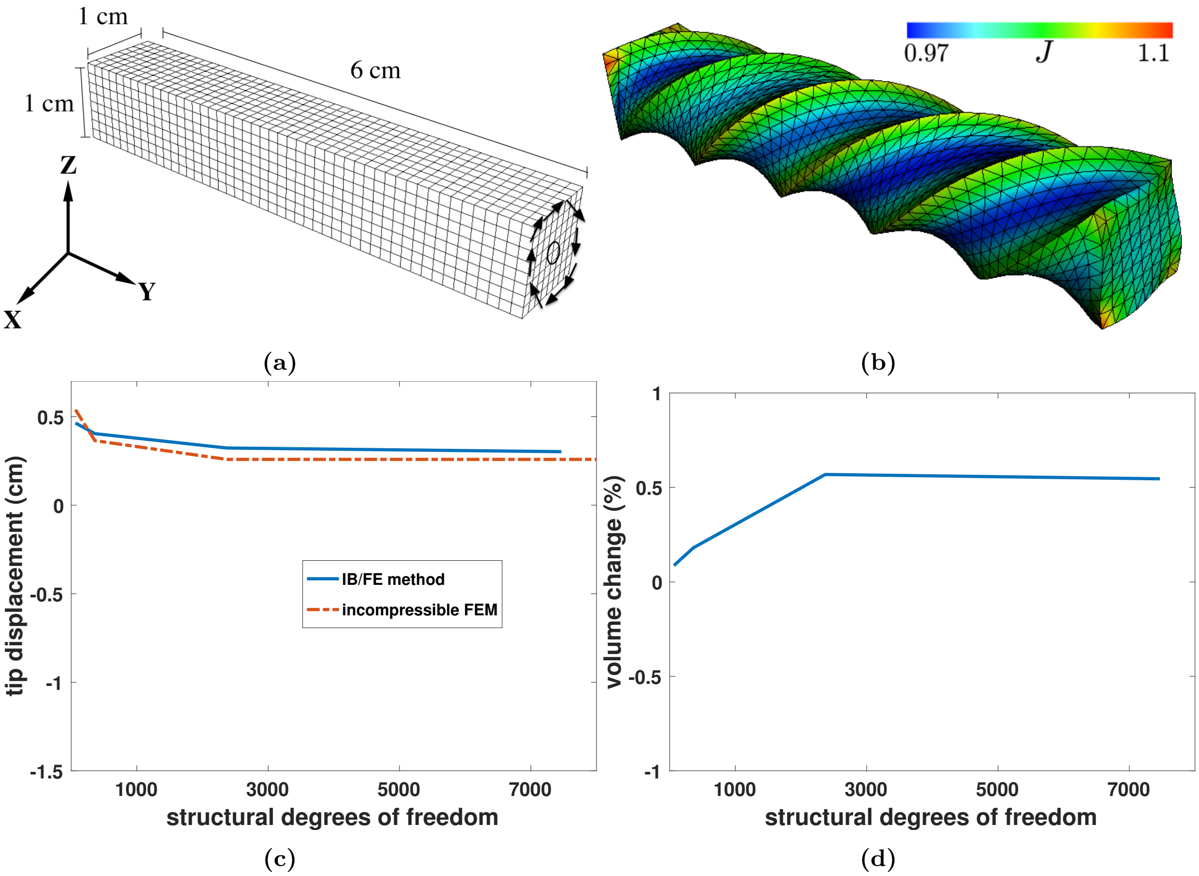


**Figure S5:** (a) Schematic of the torsion benchmark from Vadala-Roth et al. [16]. A Mooney-Rivlin material model is used, and torsion is applied to the top face of the beam while zero displacement is imposed at the opposite face. Zero traction boundary conditions were used on all the other faces, as in Bonet et al. [18]. (b) Deformation of the torsion test for a Mooney-Rivlin material model. The stabilization method results in smooth element deformation as well as excellent volume conservation. The color shows the magnitude of $J = \det(\mathbb{F})$. (c) Displacement of the tip position (the circled location in panel a) on the beam in the axial (Y) direction compared to result from using standard incompressible structural mechanics formulation. (d) Percent change in total volume; see also panel (b).

the aortic test section, 0.75 mm ($N = 192$), 0.59 mm ($N = 256$), and 0.45 mm ($N = 320$) for the porcine aortic valve, and 0.75 mm ($N = 192$, 256, and 320) with a wider range of grid-spacings for the bovine pericardial valve.

Figure S6 compares the bulk flow properties under grid refinement, indicating that our simulations are consistent under grid refinement. This suggests that the results reported in the main text are sufficiently resolved for these measurements by the resolutions considered therein. Tables S1, S2, S3, and S4 quantify the discrepancies relative to the experimental data under grid refinement, as well as the relative difference between resolutions ($N = 192$, $N = 256$, and $N = 320$). The relative differences are calculated by

$$\text{Normalized difference of } M^{N=192} \text{ and } M^{N=256} = \frac{\left\| M^{N=192} - M^{N=256} \right\|_{L^q(0,T)}}{\left\| M^{N=256} \right\|_{L^q(0,T)}}, \tag{S24}$$

$$\text{Normalized difference of } M^{N=256} \text{ and } M^{N=320} = \frac{\left\| M^{N=256} - M^{N=320} \right\|_{L^q(0,T)}}{\left\| M^{N=320} \right\|_{L^q(0,T)}}, \tag{S25}$$

in which $(0, T)$ indicates an integral over time, and $q = 2$ or $\infty$ for the $L^2$- and $L^\infty$-norms, respectively. This shows that the discrepancies in bulk flow measurements compared to the experimental data are within

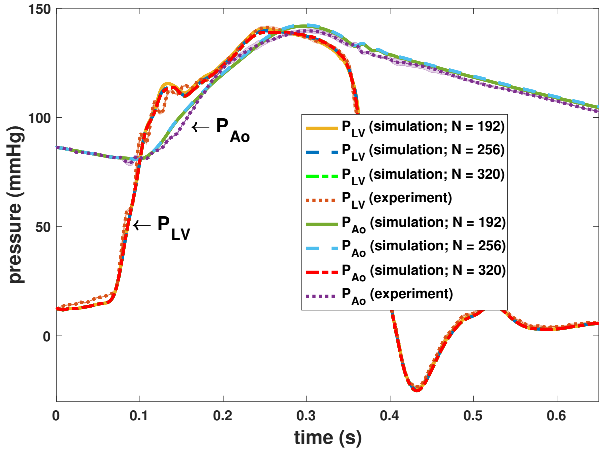


**(a)** Porcine aortic valve

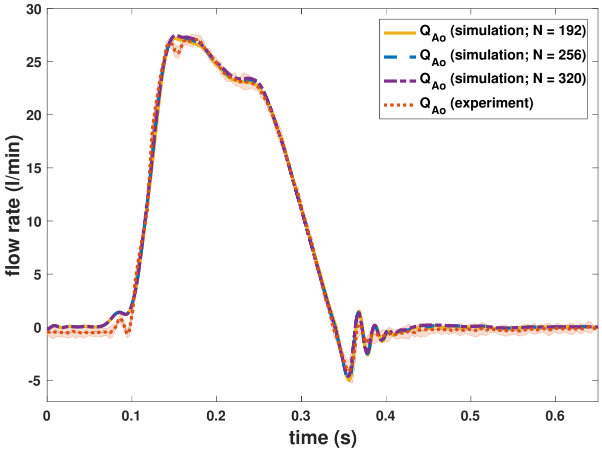


**(b)** Porcine aortic valve

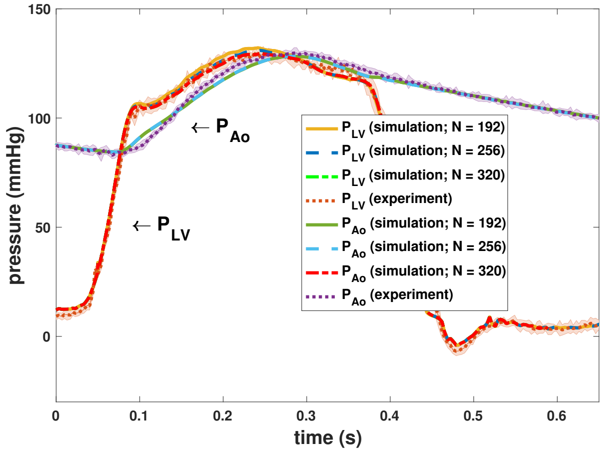


**(c)** Bovine pericardial valve

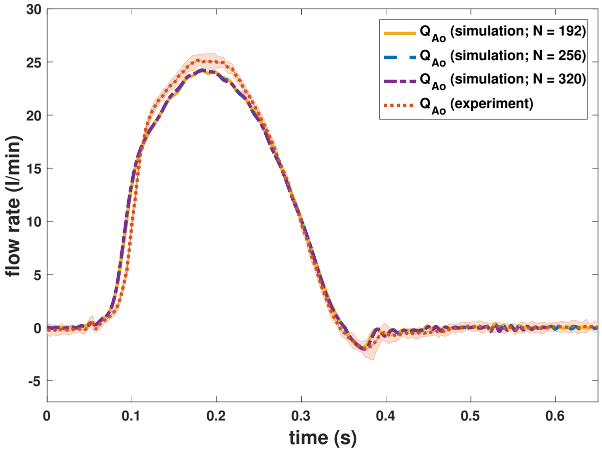


**(d)** Bovine pericardial valve

**Figure S6:** Grid refinement test corresponding to Figure 4 of the main text. The bulk flow waveforms are consistent under grid refinement.

8.7% at all resolutions. The relative differences between successive resolutions are within 1.6%, indicating consistency under grid refinement. This suggests that the bulk properties are reasonably resolved by our FSI model of the BHVs at the higher spatial resolutions ($N = 256$ and $N = 320$).

**Table S1:** Comparisons of normalized $L^2$-norms of discrepancies in the bulk measurements between simulation and experiment*

| | Porcine aortic | | | Bovine pericardial | | |
|---|---|---|---|---|---|---|
| | $N = 192$ | $N = 256$ | Relative | $N = 192$ | $N = 256$ | Relative |
| $P_{\mathrm{LV}}$ | 3.6% | 3.4% | 0.73% | 5.2% | 4.6% | 1.1% |
| $P_{\mathrm{Ao}}$ | 1.7% | 1.9% | 0.33% | 1.4% | 1.4% | 0.09% |
| $Q_{\mathrm{Ao}}$ | 5.0% | 4.7% | 1.6% | 8.4% | 8.6% | 0.71% |

* Comparisons of $L^2$-norms of the discrepancies in the bulk measurements from the simulation and experiment shown in Figure S6 under the coarser ($N = 192$) and finer ($N = 256$) resolutions, normalized by the $L^2$-norms of the measurements from the experiment. Relative indicates the $L^2$-norms of the differences between the measurements from the two resolutions, normalized by the $L^2$-norms of the measurements from the finer ($N = 256$) case.

The comparison of PDVA results is similar to that of other bulk measurements under grid refinement, as

**Table S2:** Comparisons of normalized $L^2$-norms of discrepancies in the bulk measurements between simulation and experiment*

| | Porcine aortic | | | Bovine pericardial | | |
|---|---|---|---|---|---|---|
| | $N = 256$ | $N = 320$ | Relative | $N = 256$ | $N = 320$ | Relative |
| $P_{\mathrm{LV}}$ | 3.4% | 3.4% | 0.45% | 4.6% | 4.5% | 0.67% |
| $P_{\mathrm{Ao}}$ | 1.9% | 1.9% | 0.21% | 1.4% | 1.4% | 0.004% |
| $Q_{\mathrm{Ao}}$ | 4.7% | 4.8% | 0.74% | 8.6% | 8.7% | 0.43% |

* Comparisons of $L^2$-norms of the discrepancies in the bulk measurements from the simulation and experiment shown in Figure S6 under the finer ($N = 256$) and finest ($N = 320$) resolutions, normalized by the $L^2$-norms of the measurements from the experiment. Relative indicates the $L^2$-norms of the differences between the measurements from the two resolutions, normalized by the $L^2$-norms of the measurements from the finer ($N = 320$) case.

**Table S3:** Comparisons of normalized $L^\infty$-norms of discrepancies in the bulk measurements between simulation and experiment*

| | Porcine aortic | | | Bovine pericardial | | |
|---|---|---|---|---|---|---|
| | $N = 192$ | $N = 256$ | Relative | $N = 192$ | $N = 256$ | Relative |
| $P_{\mathrm{LV}}$ | 8.5% | 8.6% | 1.6% | 15.6% | 14.4% | 1.7% |
| $P_{\mathrm{Ao}}$ | 4.3% | 4.5% | 0.42% | 3.5% | 3.6% | 0.17% |
| $Q_{\mathrm{Ao}}$ | 11.6% | 9.5% | 2.4% | 19.2% | 20.2% | 1.4% |

* Comparisons of $L^\infty$-norms of the discrepancies in the bulk measurements from the simulation and experiment shown in Figure S6 between the coarser ($N = 192$) and finer ($N = 256$) resolutions, normalized by the $L^\infty$-norms of the measurements from the experiment. Relative indicates the $L^\infty$-norms of the differences between the measurements from the two resolutions, normalized by the $L^\infty$-norms of the measurements from the finer ($N = 256$) case.

**Table S4:** Comparisons of normalized $L^\infty$-norms of discrepancies in the bulk measurements between simulation and experiment*

| | Porcine aortic | | | Bovine pericardial | | |
|---|---|---|---|---|---|---|
| | $N = 256$ | $N = 320$ | Relative | $N = 256$ | $N = 320$ | Relative |
| $P_{\mathrm{LV}}$ | 8.6% | 8.6% | 1.5% | 14.4% | 14.1% | 1.1% |
| $P_{\mathrm{Ao}}$ | 4.5% | 4.5% | 0.31% | 3.6% | 3.6% | 0.001% |
| $Q_{\mathrm{Ao}}$ | 9.5% | 9.2% | 2.2% | 20.2% | 20.8% | 0.6% |

* Comparisons of $L^\infty$-norms of the discrepancies in the bulk measurements from the simulation and experiment shown in Figure S6 between the finer ($N = 256$) and finest ($N = 320$) resolutions, normalized by the $L^\infty$-norms of the measurements from the experiment. Relative indicates the $L^\infty$-norms of the differences between the measurements from the two resolutions, normalized by the $L^\infty$-norms of the measurements from the finer ($N = 320$) case.

observed in Figure S6.

Figures S8 and S9 also give a qualitative sense of model consistency under grid refinement. Although Figures S8 and S9 indicate that additional small-scale flow features are generated by the model at higher spatial resolutions, the large scale flow features are consistent between grid resolutions.

We also observe that the time-averaged flow fields (Eq. S26) as well as the flow profiles (Figures S10 and S11) are in reasonable agreement between different spatial resolutions. The mean flow is determined via

$$\overline{u}_i = \frac{1}{T_2 - T_1} \int_{T_1}^{T_2} u_i(\boldsymbol{x}, t)\ \mathrm{d}t, \quad i = 1, 2, 3, \tag{S26}$$

We consider steady inflow conditions at two different flow rates: 100% and 50% of the maximum flow rate from the fully dynamic simulations. In particular, we find that the difference between $N = 256$ and $N = 320$

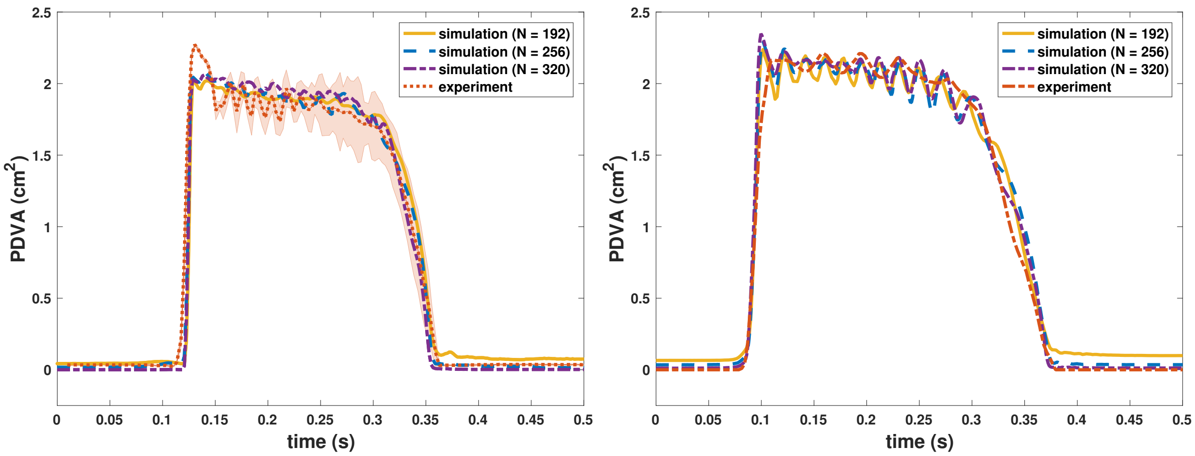


**(a)** Porcine aortic valve

**(b)** Bovine pericardial valve

**Figure S7:** Grid refinement test corresponding to Figure 5 of the main text. The PDVA results are consistent under grid refinement. This result is in agreement with the consistency under grid refinement demonstrated in Figure S6d.

cases are small, and we conclude that $N = 256$ results are reasonably well resolved for large-scale flow measures.

We observe that the turbulence kinetic energy (Eq. S27) as well as its profiles for each bovine pericardial valve case (Figures S12 and S13) still show some differences between different spatial resolutions. Turbulence kinetic energy is defined by

$$TKE \equiv \frac{1}{2}\overline{u_i' u_i'}, \tag{S27}$$

in which $u_i' = u_i - \overline{u}_i$ is the fluctuating velocity. We again consider steady inflow conditions at two different flow rates: 100% and 50% of the maximum flow rate. At the maximum flow rate, the axial flow fields agree to approximately 1.6% in the $L^2$ norm for $N = 256$ and $N = 320$, with peak velocities differing by approximately 7%. At 50% of maximum flow, the flow fields agree to within approximately 1.1% in the $L^2$ norm, with peak velocities agreeing to within approximately 4%. The differences are larger for the TKE, with $L^2$ differences on the order of 10% and maximum TKE values differing by 17.5% at the maximum flow rate but to within approximately 1.5% at 50% of the maximum flow rate. The mean flow and TKE profiles are in reasonable agreement and appear to be converging under grid refinement. Although we find that the differences between $N = 256$ and $N = 320$ are not large, these results suggest that we are able to resolve the large-scale flow features, but higher spatial resolution is clearly needed to fully resolve the flow fields.

With steady inflow conditions, as in the pulsatile cases, the computed values of PDVA yielded by the model are in good agreement at the different grid spacings (data not shown). It is apparent, however, that the peak flow velocities for the coarser cases are larger than those of the finer cases. The observed differences in the peak flow velocities are a consequence of the numerical scheme that is used to couple the Lagrangian and Eulerian variables in the present IB method. Specifically, the use of regularized delta functions in the Lagrangian-Eulerian coupling operators causes the "effective" sizes of the Lagrangian structures to be larger than their "actual" sizes, with a length scale that is related to the regularization width of the delta function. In practice, the width of the regularized delta function is tied to the Cartesian grid spacing. This results in an effective narrowing of the flow path that is on the order of a few Cartesian grid cell widths, which is more pronounced on coarser grids. The effect of this narrowing is apparent in Figures S10 and S11 and results in higher flow velocities at the same volumetric flow rates. This effect also diminishes under grid refinement, as observed in the grid convergence study.

Figures S14 and S15 qualitatively compare the leaflet kinematics for $N = 192$, $N = 256$, and $N = 320$ during valve opening and closure for the porcine aortic valve and bovine pericardial valve, respectively. These results indicate that the computed structural deformations are consistent under grid refinement.

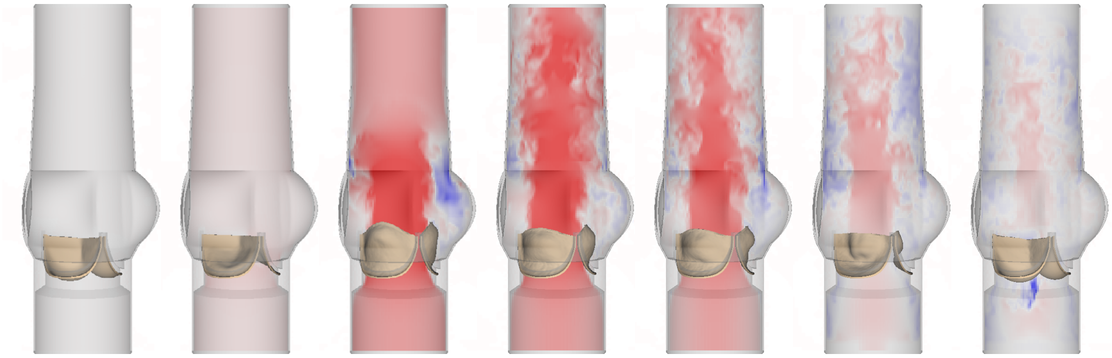

**(a)** $N = 192$

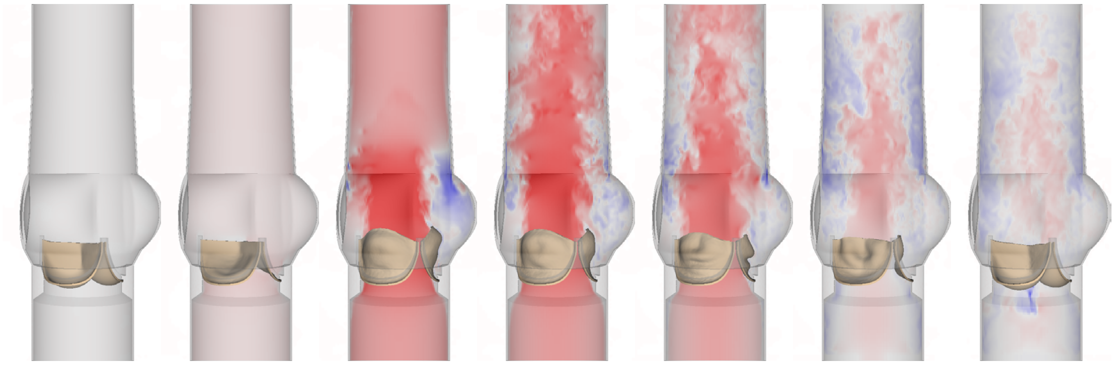

**(b)** $N = 256$

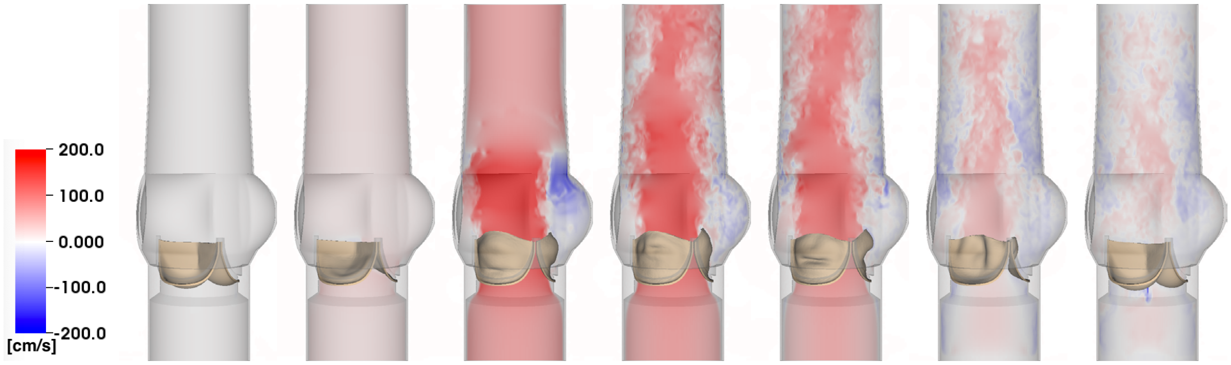


**(c)** $N = 320$

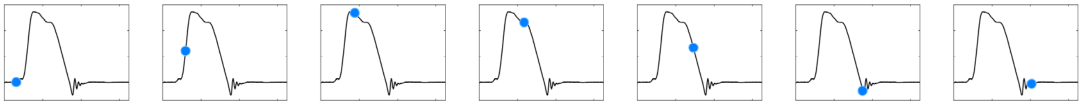

**Figure S8:** Grid refinement test corresponding to Figure 7a of the main text. Although additional small-scale flow features are generated by the model at higher spatial resolutions, the large scale flow features are consistent between grid resolutions.

Comparing the stress distributions of the leaflets in two grid resolutions (Figures S16 and S17) also confirms that the stress distributions are consistent under grid refinement.

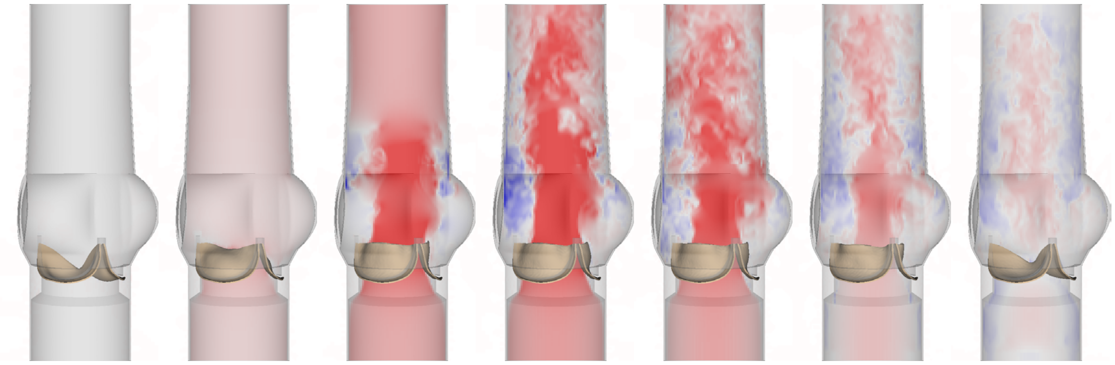

**(a)** $N = 192$

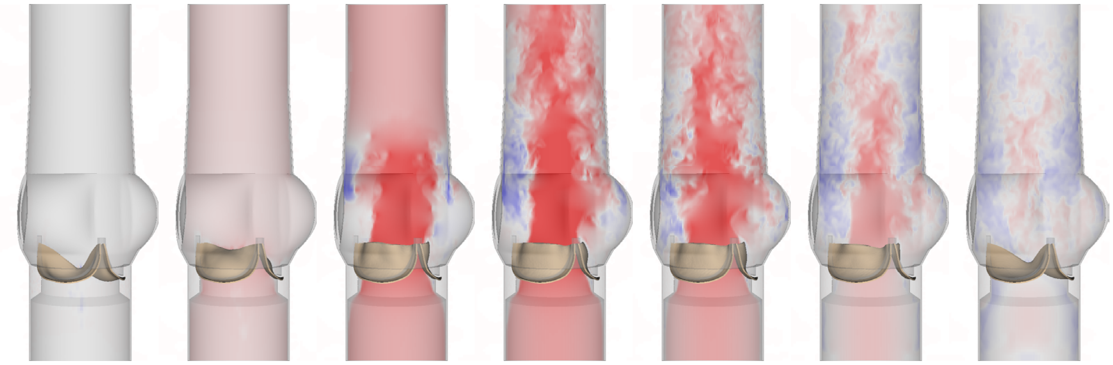

**(b)** $N = 256$

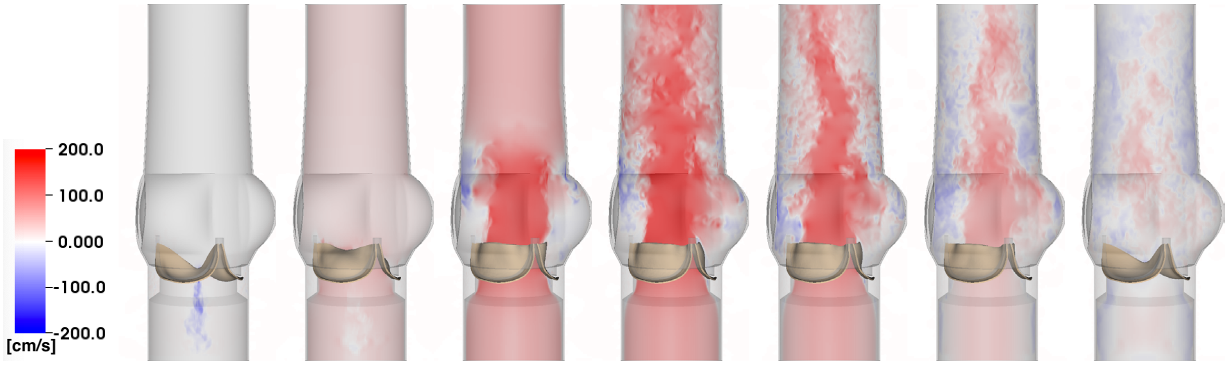


**(c)** $N = 320$

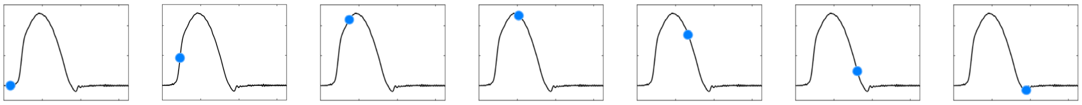

**Figure S9:** Grid refinement test corresponding to Figure 7b of the main text. Although additional small-scale flow features are generated by the model at higher spatial resolutions, the large scale flow features are consistent between grid resolutions.

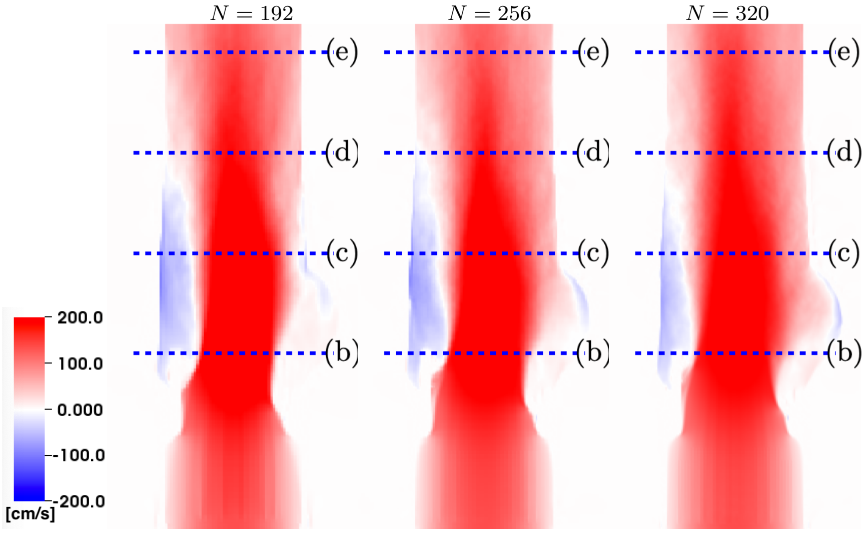


**(a)** Axial velocity ($u_y$) fields

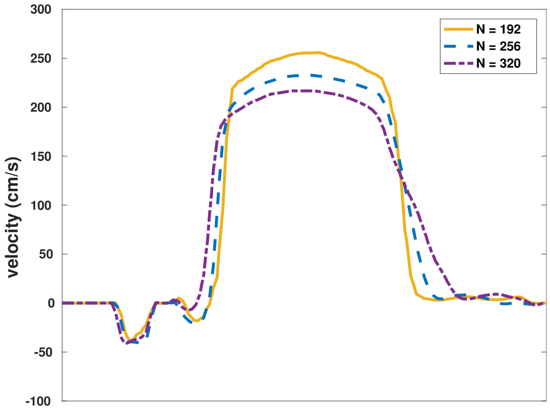


**(b)**

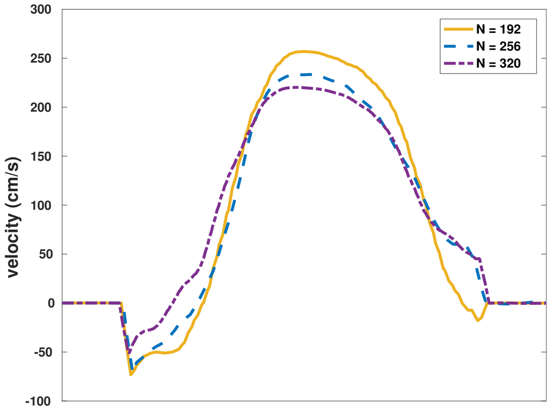


**(c)**

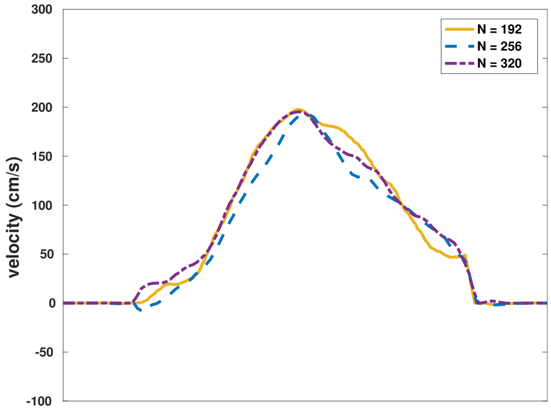


**(d)**

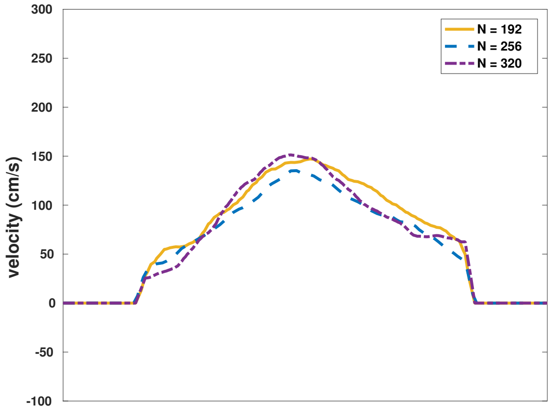


**(e)**

**Figure S10:** (a) Cross-section view of time-averaged flow fields and (b)–(e) flow profiles at different locations downstream of the valve for the bovine pericardial valve model. We use steady flow at the maximum flow rate. In panel (a), the color shows the time-averaged axial velocity through the aortic test section at the center plane, with red indicating forward flow and blue indicating reverse flow. Blue dashed lines show where the profiles shown in panels (b)–(e) are obtained. The axial flow fields agree to approximately 1.6% in the $L^2$ norm for $N = 256$ and $N = 320$, with peak velocities differing by approximately 7%.

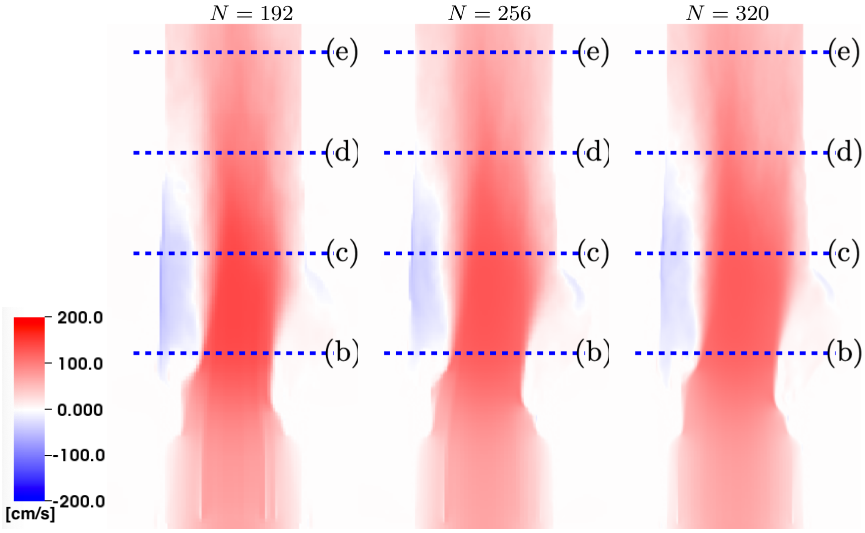


**(a)** Axial velocity ($u_y$) fields

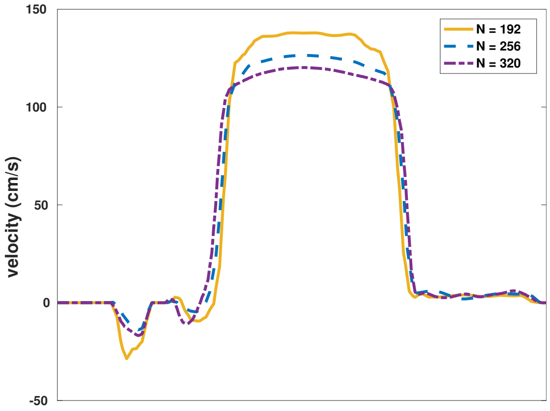


**(b)**

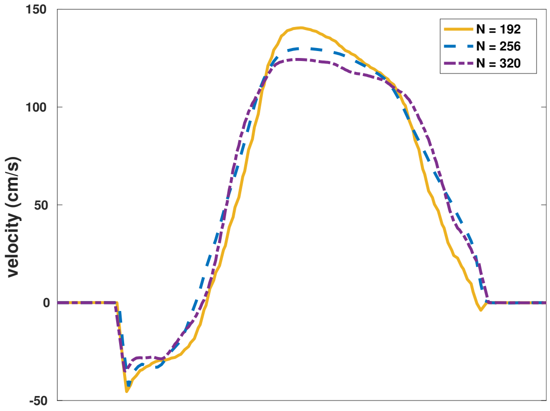


**(c)**

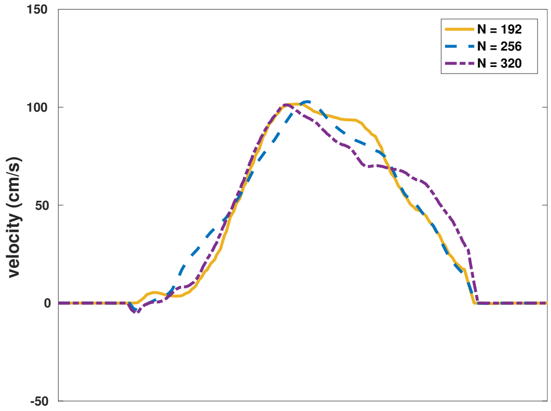


**(d)**

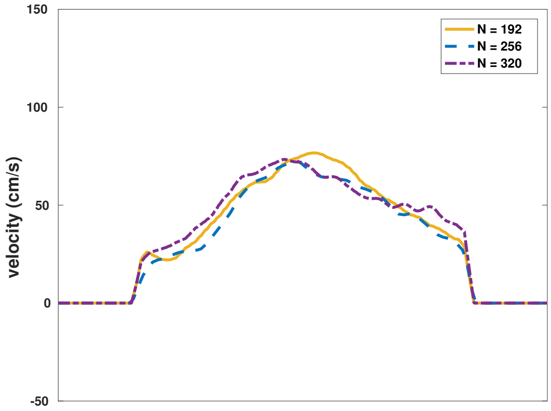


**(e)**

**Figure S11:** (a) Cross-section view of time-averaged flow fields and (b)–(e) flow profiles at different locations downstream of the valve for the bovine pericardial valve model. We use steady flow at 50% of the maximum flow rate. In panel (a), the color shows the time-averaged axial velocity through the aortic test section at the center plane, with red indicating forward flow and blue indicating reverse flow. Blue dashed lines show where the profiles shown in panels (b)–(e) are obtained. The flow fields agree to within approximately 1.1% in the $L^2$ norm, with peak velocities agreeing to within approximately 4%.

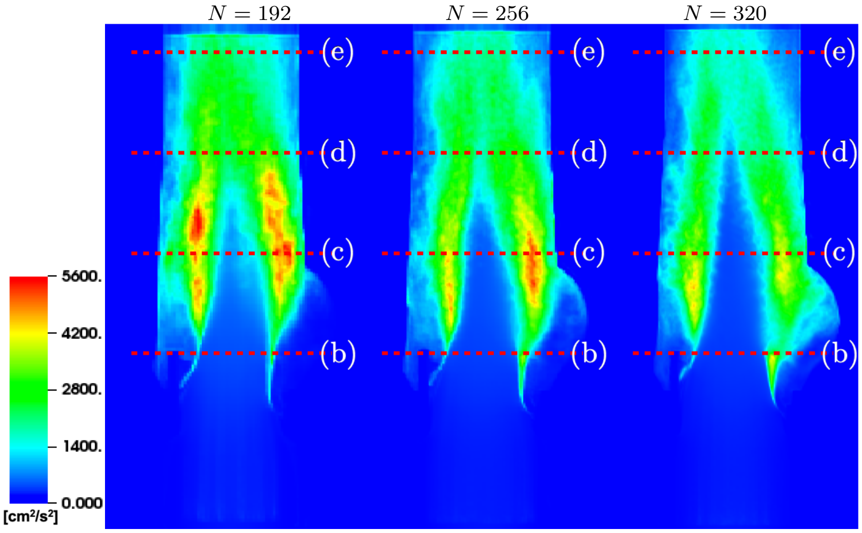


**(a)** TKE fields

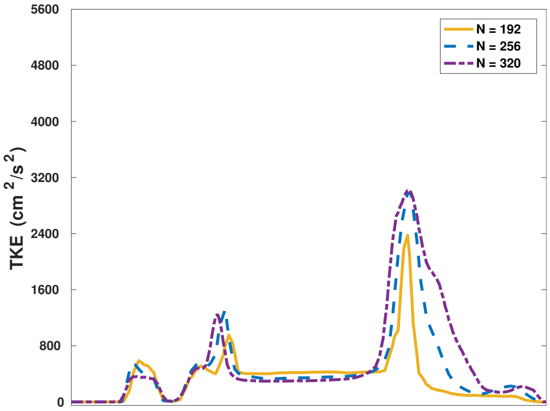


**(b)**

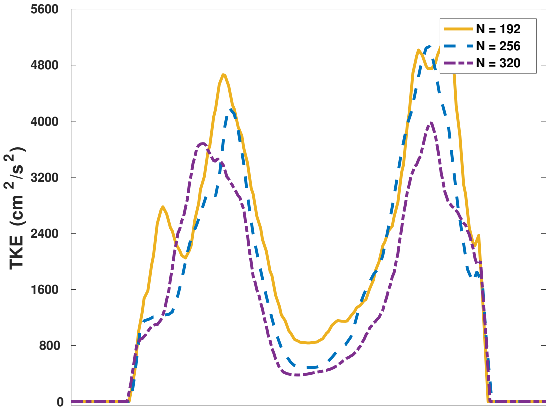


**(c)**

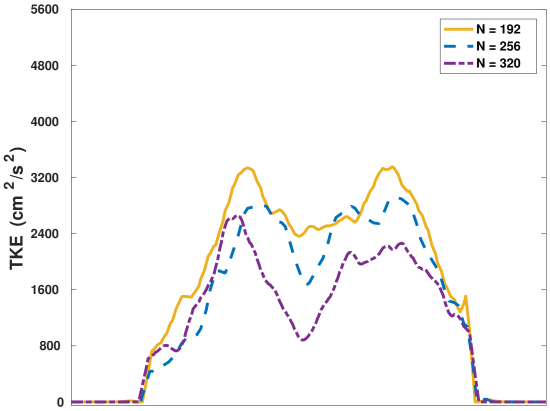


**(d)**

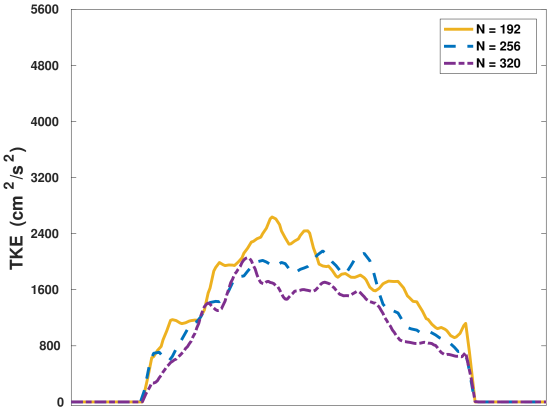


**(e)**

**Figure S12:** (a) Cross-section view of turbulence kinetic energy (TKE) fields and (b)–(e) TKE profiles at different locations downstream of the valve for the bovine pericardial valve model. We use steady flow at the maximum flow rate. In panel (a), the color shows the magnitude of TKE through the aortic test section at the center plane. Red dashed lines show where the profiles shown in panels (b)–(e) are obtained. The $L^2$ differences are on the order of 10% between $N = 256$ and $N = 320$, with peak values of TKE differing by approximately 17.5%.

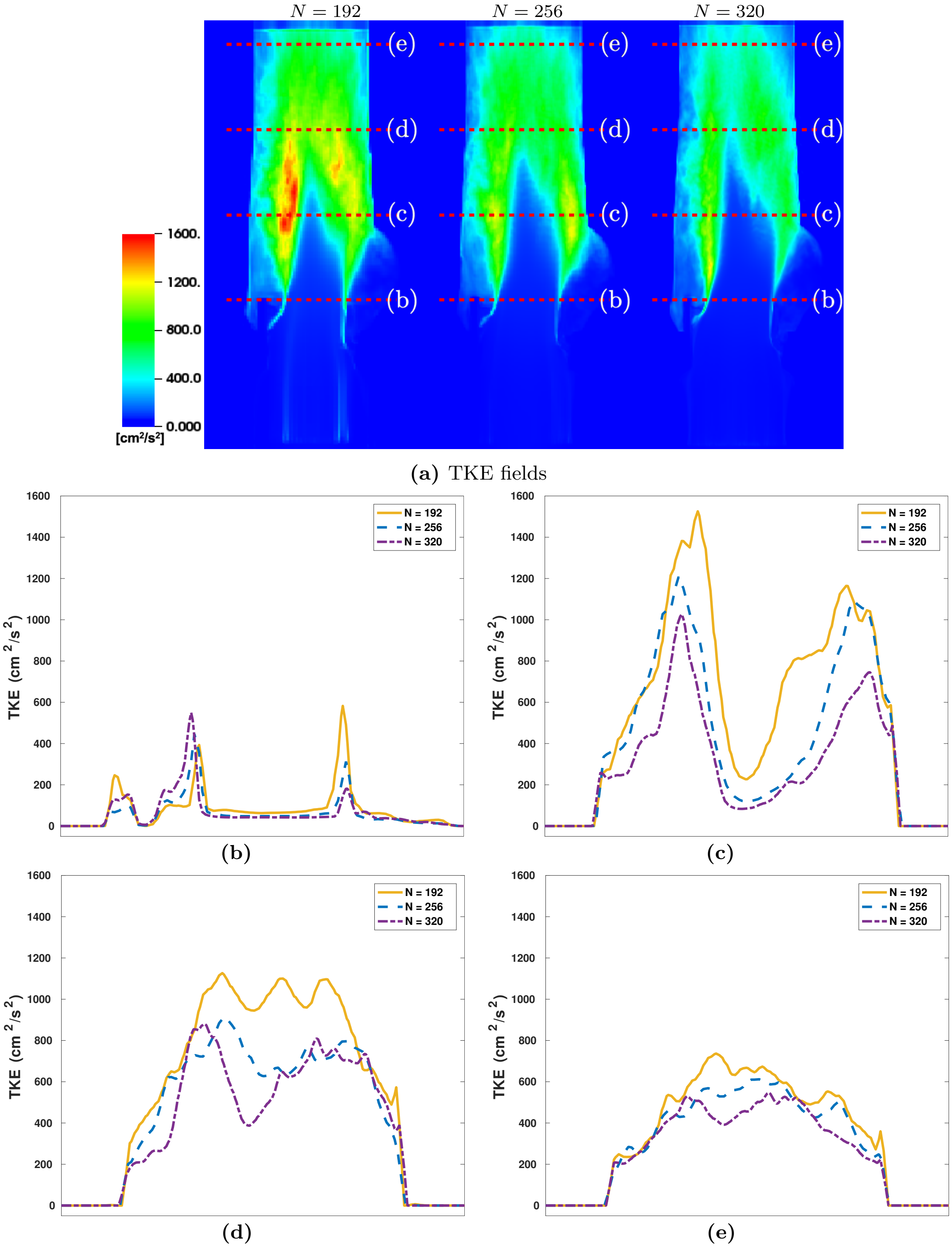


**Figure S13:** (a) Cross-section view of turbulence kinetic energy (TKE) fields and (b)–(e) TKE profiles at different locations downstream of the valve for the bovine pericardial valve model. We use steady flow at 50% of the maximum flow rate. In panel (a), the color shows the magnitude of TKE through the aortic test section at the center plane. Red dashed lines show where the profiles shown in panels (b)–(e) are obtained. The $L^2$ differences are on the order of 10% between $N = 256$ and $N = 320$, with peak values of TKE differing by approximately 1.5%.

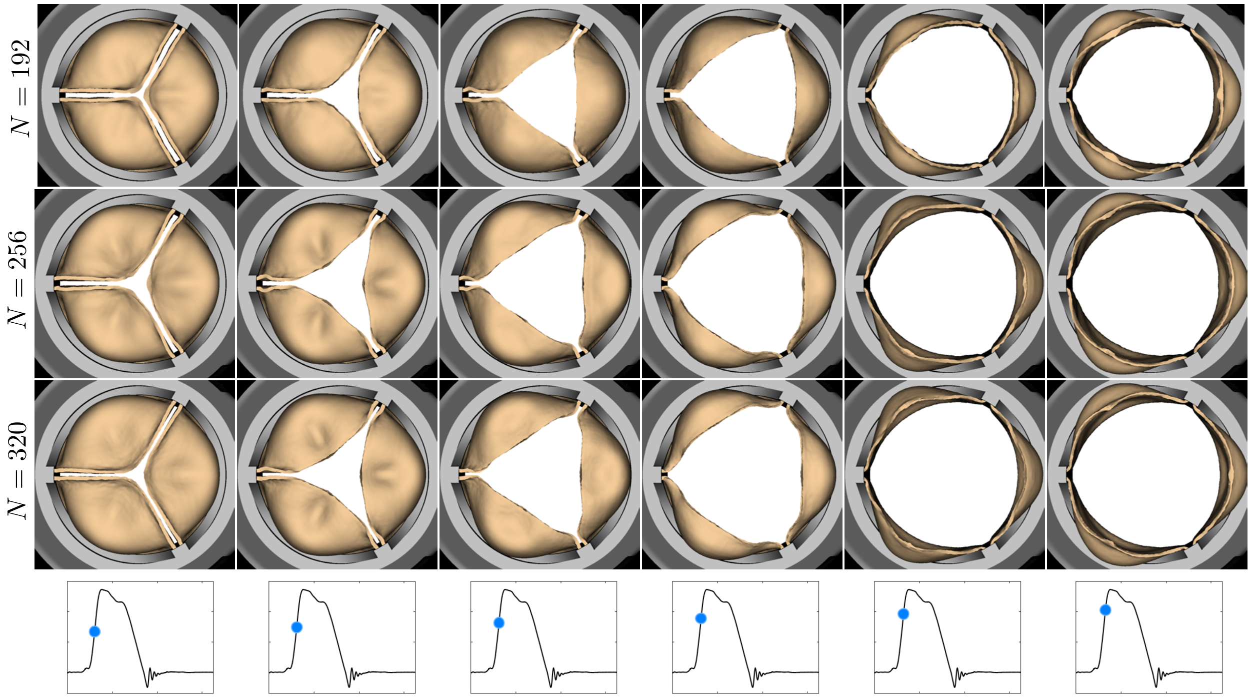


**(a)**

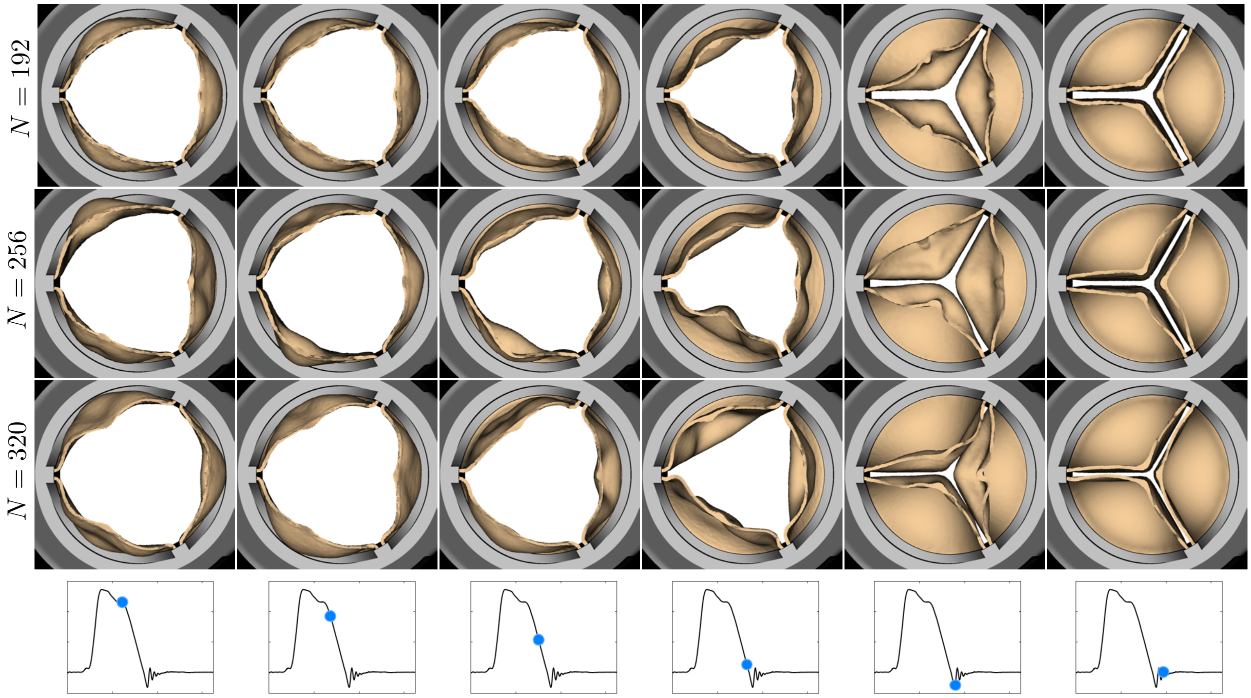


**(b)**

**Figure S14:** Leaflet kinematics of the porcine aortic valve during (a) valve opening and (b) valve closure under grid refinement. *Top panels*: $N = 192$, *middle panels*: $N = 256$, and *bottom panels*: $N = 320$. The time increment between frames in panel (a) is 1.92 ms, and the time increment between frames in panel (b) is 9.6 ms.

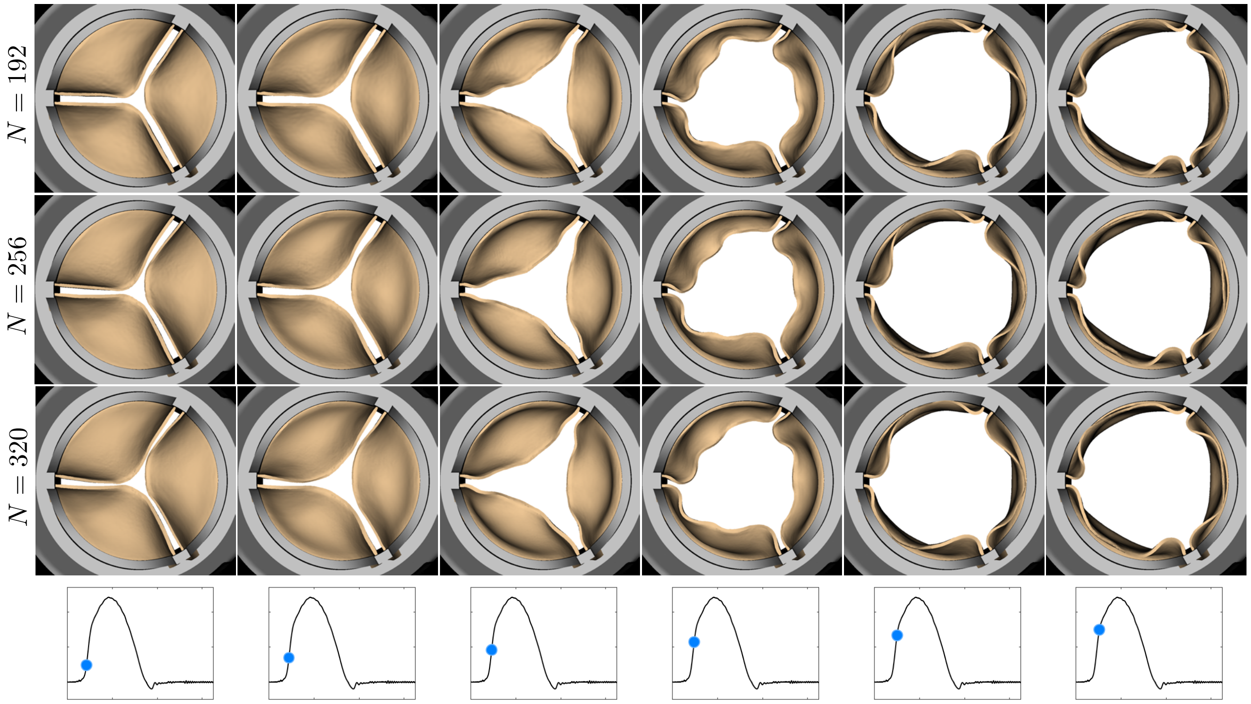


**(a)**

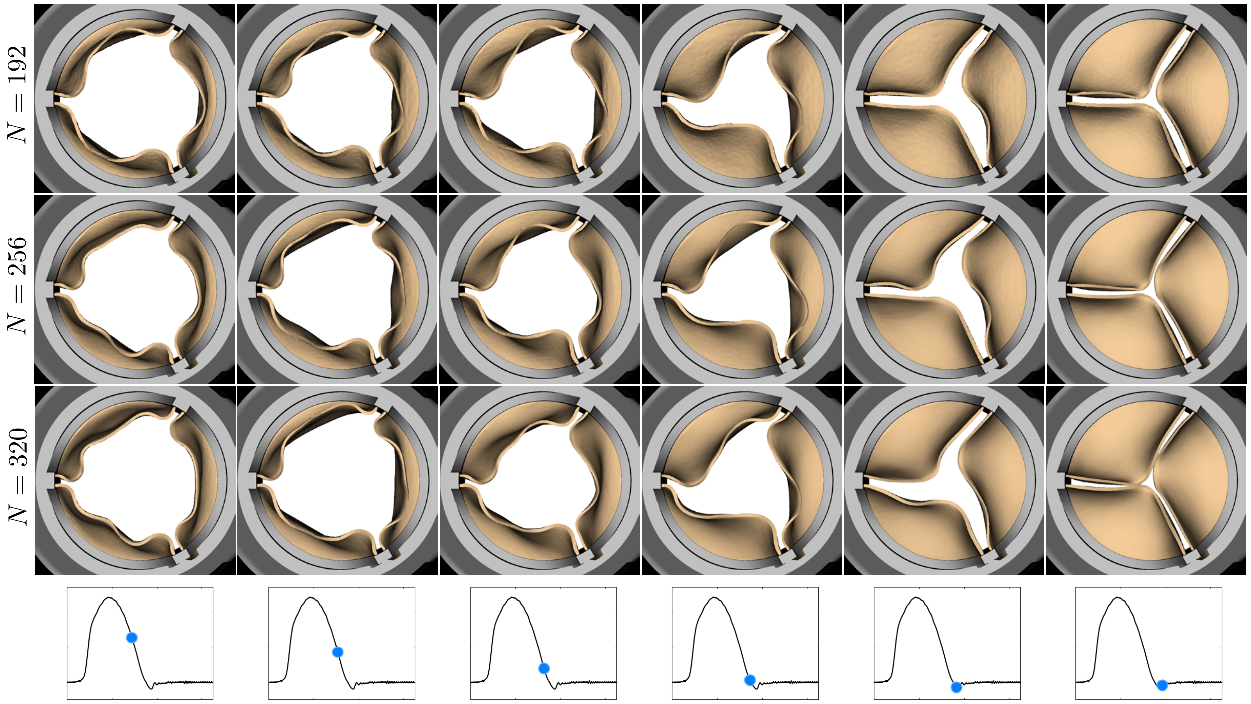


**(b)**

**Figure S15:** Leaflet kinematics of the bovine pericardial valve during (a) valve opening and (b) valve closure under grid refinement. *Top panels*: $N = 192$, *middle panels*: $N = 256$, and *bottom panels*: $N = 320$. The time increment between frames in panel (a) is 3.84 ms, and the time increment between frames in panel (b) is 19.2 ms.

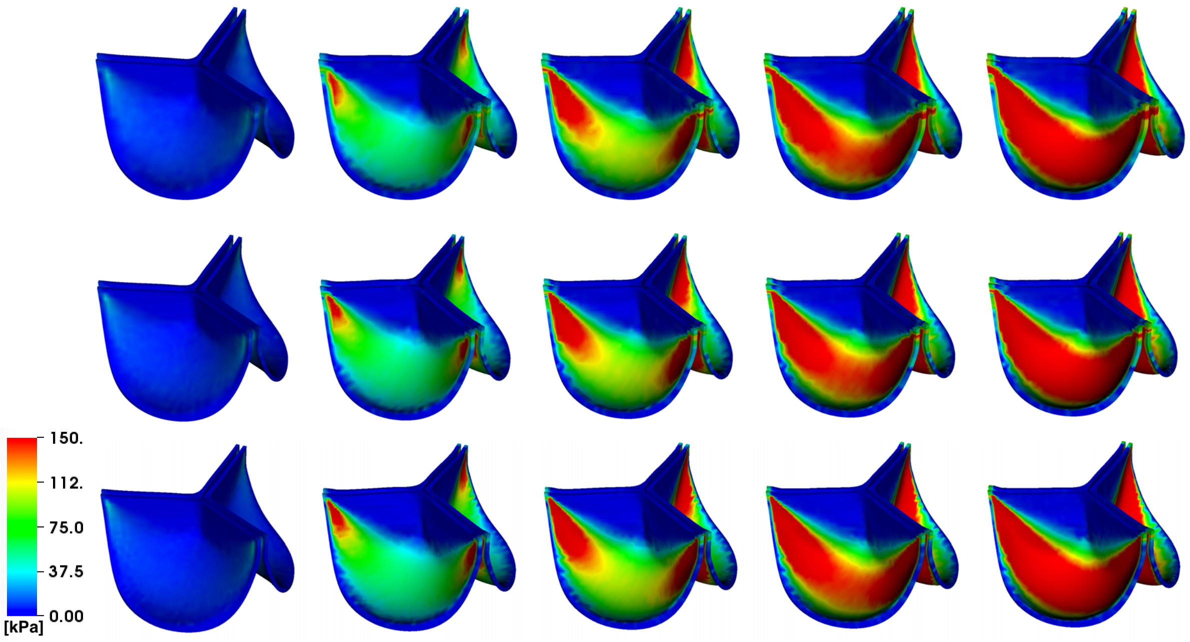


**(a)**

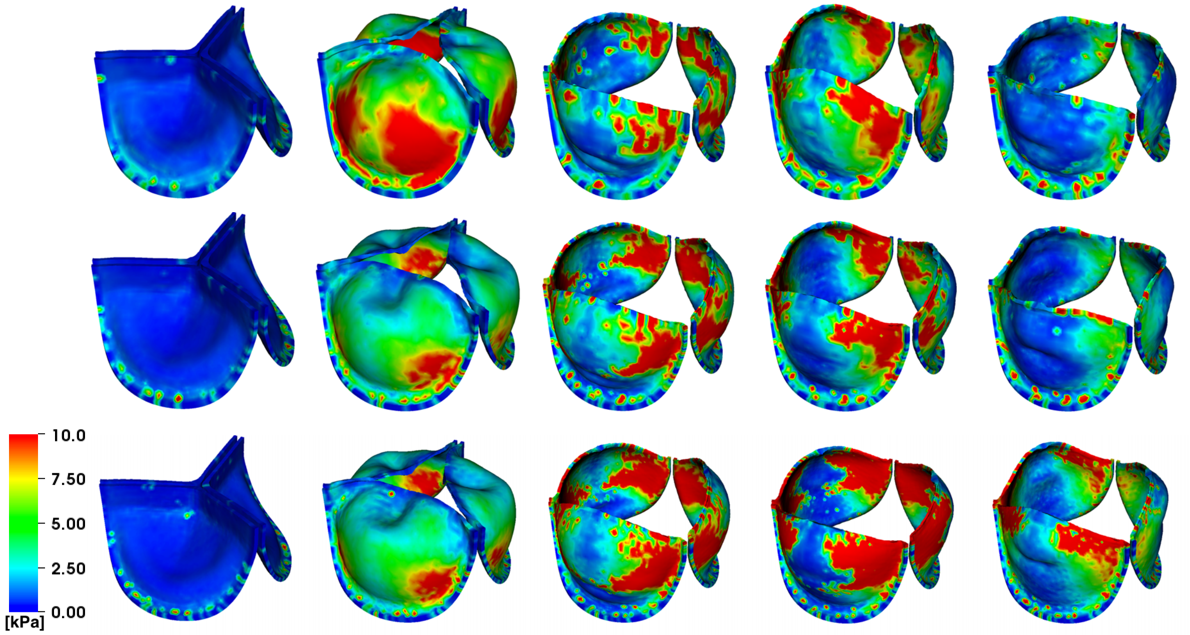


**(b)**

**Figure S16:** von Mises stress (kPa) on the porcine aortic valve during (a) valve closure and (b) valve opening under grid refinement. *Top panels*: $N = 192$, *middle panels*: $N = 256$, and *bottom panels*: $N = 320$ for both cases. The time increment between frames in panel (a) is 30.72 ms, and the time increment between frames in panel (b) is 11.52 ms.

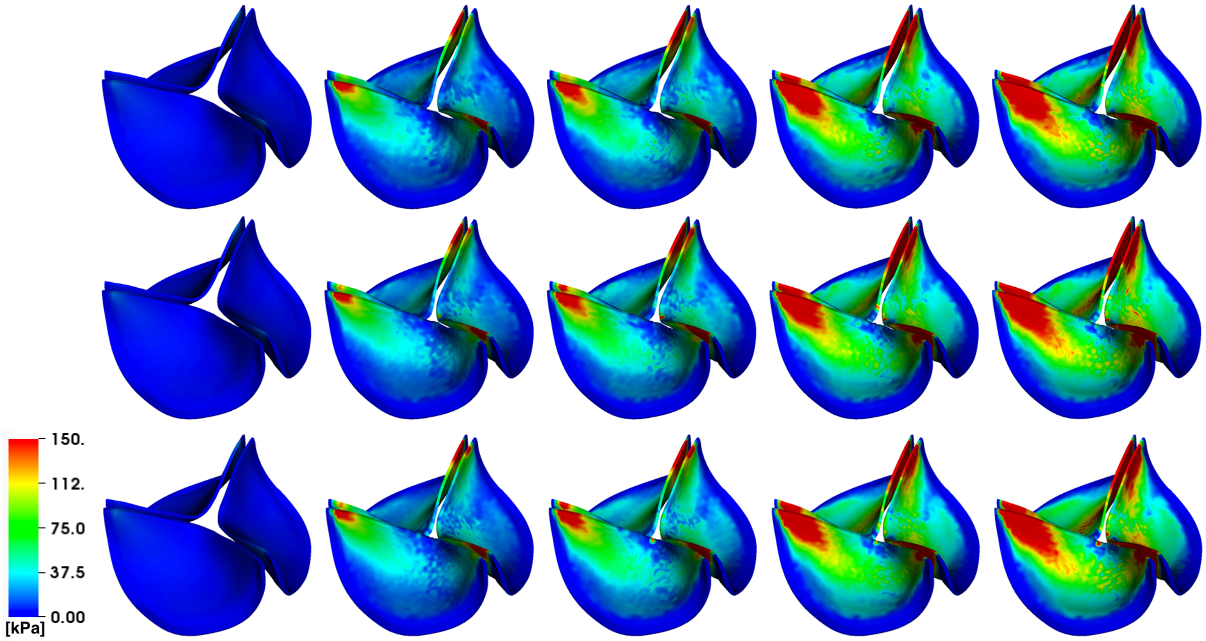


**(a)**

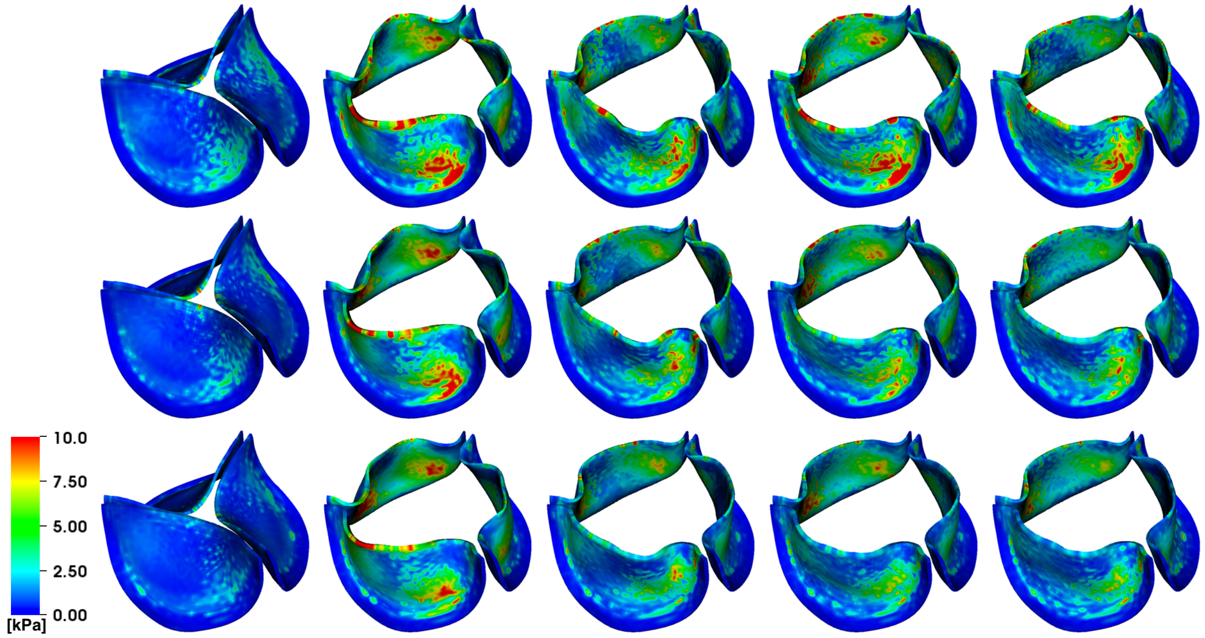


**(b)**

**Figure S17:** von Mises stress (kPa) on the porcine aortic valve during (a) valve closure and (b) valve opening under grid refinement. *Top panels*: $N = 192$, *middle panels*: $N = 256$, and *bottom panels*: $N = 320$ for both cases. The time increment between frames in panel (a) is 30.72 ms, and the time increment between frames in panel (b) is 11.52 ms.

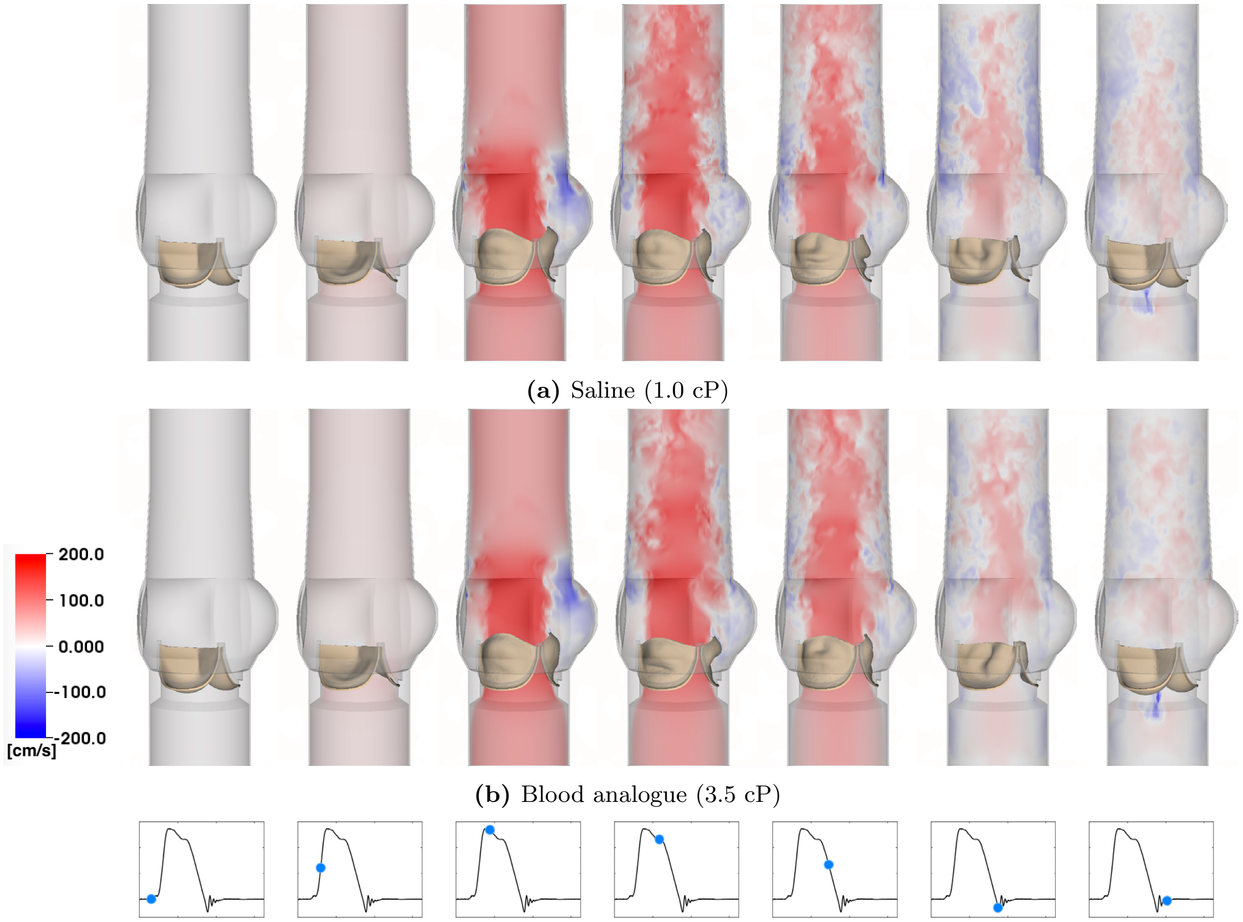


**(a)** Saline (1.0 cP)

**(b)** Blood analogue (3.5 cP)

**Figure S18:** Cross-section view of simulated flow patterns for the porcine aortic valve model using (a) saline with density 1.0 g cm$^{-3}$ and viscosity 1.0 cP versus (b) Newtonian blood analogue with density 1.0 g cm$^{-3}$ and viscosity 3.5 cP. The color shows the axial velocity through the aortic test section at the center plane, with red indicating forward flow and blue indicating reverse flow. (a) $Re_{\text{peak}} = 20{,}576$. (b) $Re_{\text{peak}} = 5{,}879$. The time increment between frames is 57.6 ms.

# G Comparison Between Saline and Newtonian Blood Analogue Test Fluids

The goal of this study is to present our FSI models of bioprosthetic heart valves in the in vitro pulse duplicator system in the same setting as the completed experiments, which use saline as the test fluid. Even so, we performed additional simulations with a Newtonian blood analogue fluid with density 1.0 g cm$^{-3}$ and viscosity 3.5 cP to show that our FSI models can be used in such flow regime without numerical instabilities (Figures S18 and S19). We are also interested in understanding the influence of using saline as compared to a more realistic blood analogue fluid in in vitro testing. To compare the predictions of the model for different test fluids, we perform simulations at the finer resolution ($N = 256$) for both pulsatile and steady mean simulations. For the comparison of the steady mean simulation, we only show the results for the bovine pericardial valve case. The porcine aortic valve model yields similar results and is not shown.

Our simulations indicate that the large scale flow structures are very similar between saline and the blood analogue fluid (Figures S18, S19, and S20). However, we observed differences in the small-scale turbulent flow structures present between saline and blood analogue fluid case (Figure S21), especially in the maximum steady flow case (Figure S21a). This is expected because of the larger Reynolds number associated with saline compared to the blood analogue.

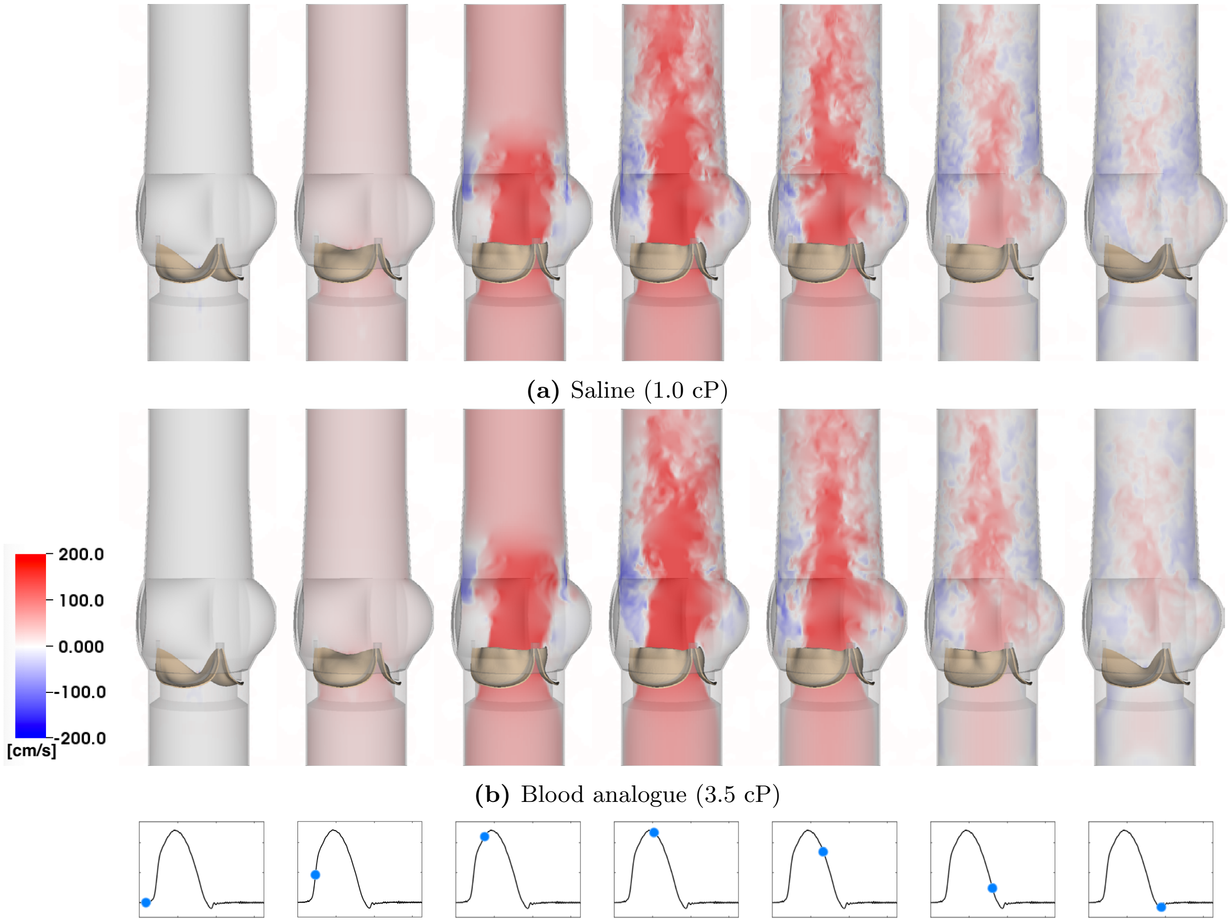


**(a)** Saline (1.0 cP)

**(b)** Blood analogue (3.5 cP)

**Figure S19:** Cross-section view of simulated flow patterns for the bovine pericardial valve model using (a) saline with density 1.0 g cm$^{-3}$ and viscosity 1.0 cP versus (b) Newtonian blood analogue with density 1.0 g cm$^{-3}$ and viscosity 3.5 cP. The color shows the axial velocity through the aortic test section at the center plane, with red indicating forward flow and blue indicating reverse flow. (a) $Re_{\text{peak}} = 19{,}330$. (b) $Re_{\text{peak}} = 5{,}523$. The time increment between frames is 57.6 ms.

Figures S22 and S23 show that the leaflet kinematics for saline versus the blood analogue fluid are similar because the leaflet kinematics are driven mostly by large-scale flow features.

# H Simulation Environment

For $N = 256$, FSI simulations take about six days to compute a full cardiac cycle using 64 processor cores on *Dogwood* cluster managed by University of North Carolina Information Technology Services Research Computing. In this study, we focus on modeling aspects rather than parallel performance analysis. There is currently an ongoing effort to improve the parallel performance of the IBAMR software library, and we aim to report on these efforts, and their impacts on models like those described herein, in the future.

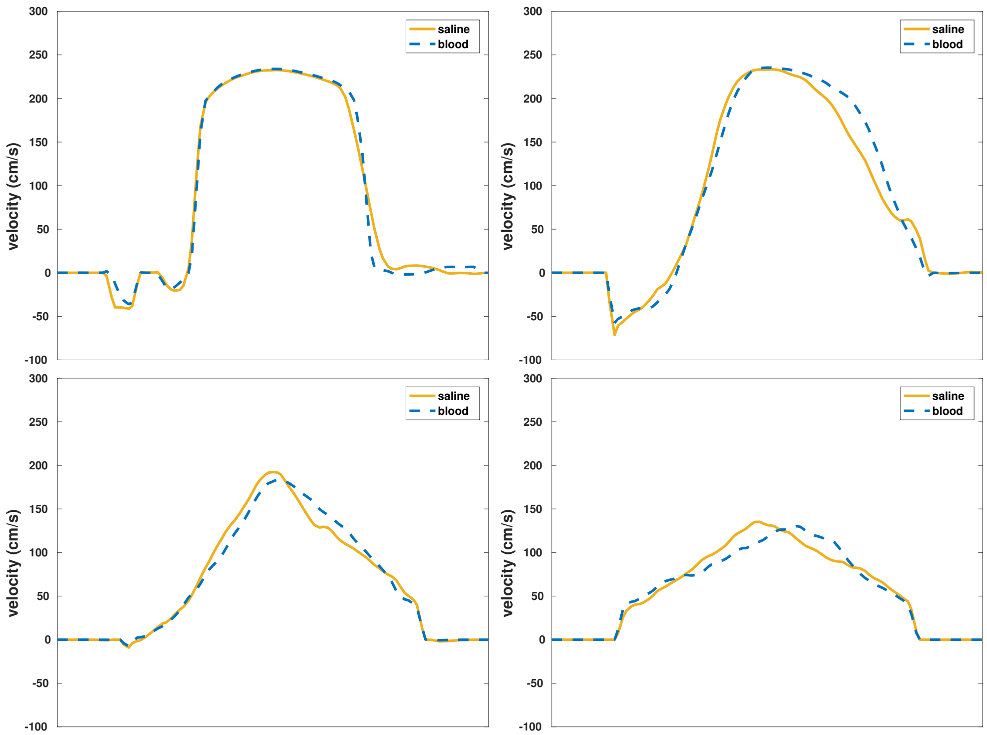


**(a)** Maximum steady flow rate

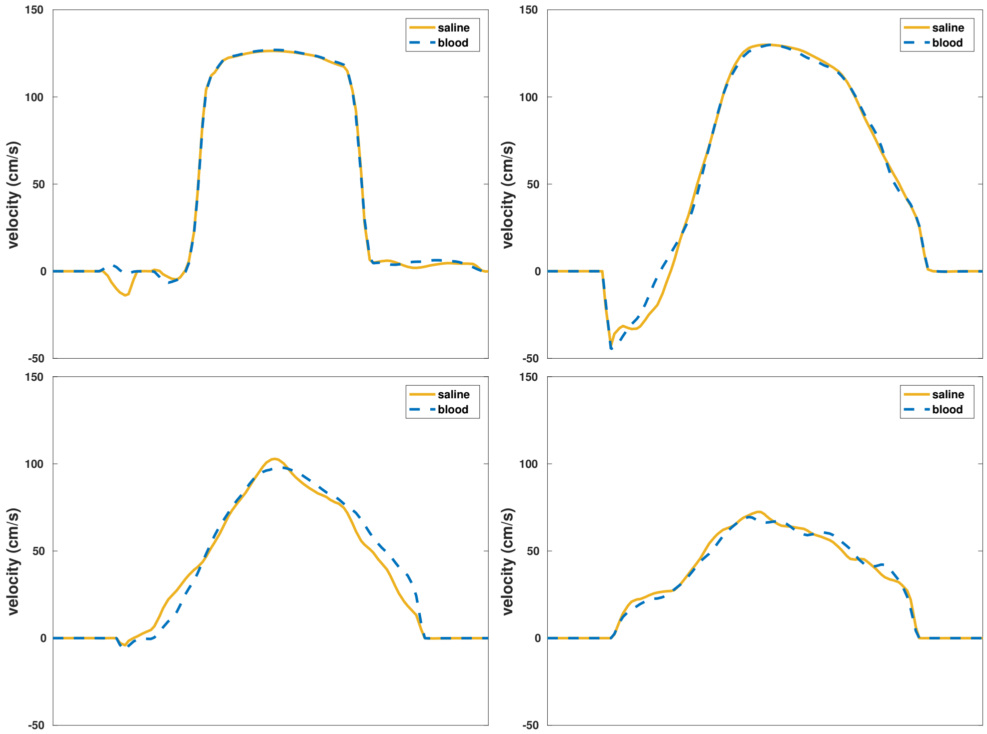


**(b)** 50% maximum steady flow rate

**Figure S20:** (a) Cross-section view of time-averaged axial velocity profiles at different locations downstream of the valve for the bovine pericardial valve model. We use steady flow at (a) the maximum and (b) 50% of the maximum flow rate. Flow profiles are show at the same locations as indicated in Figures S10 and S11.

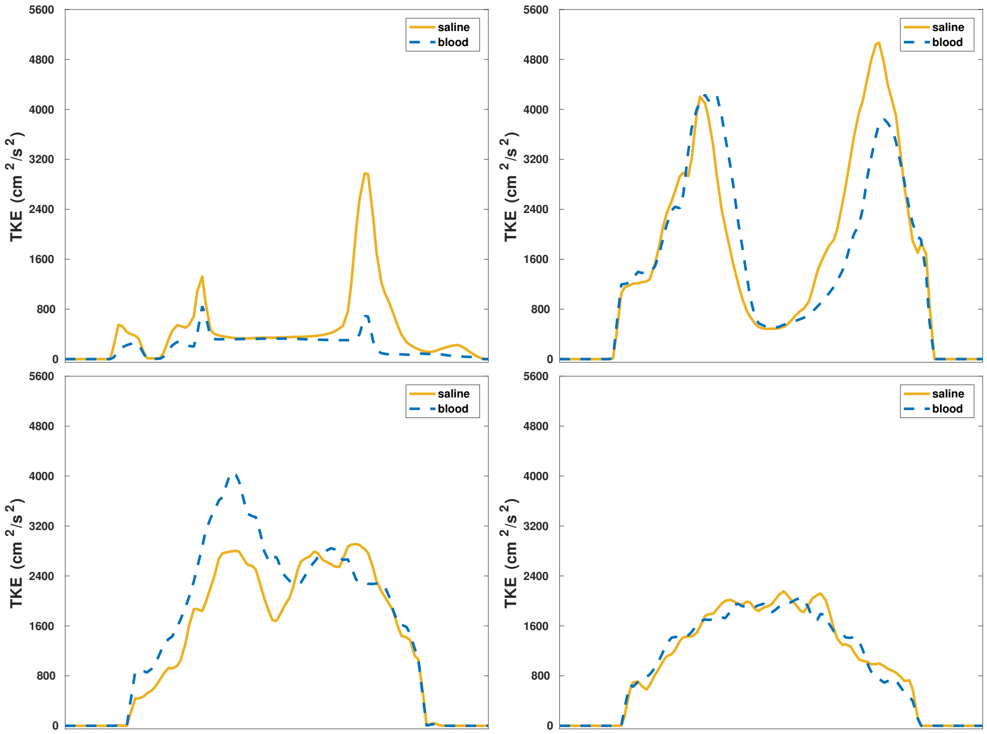


**(a)** Maximum steady flow rate

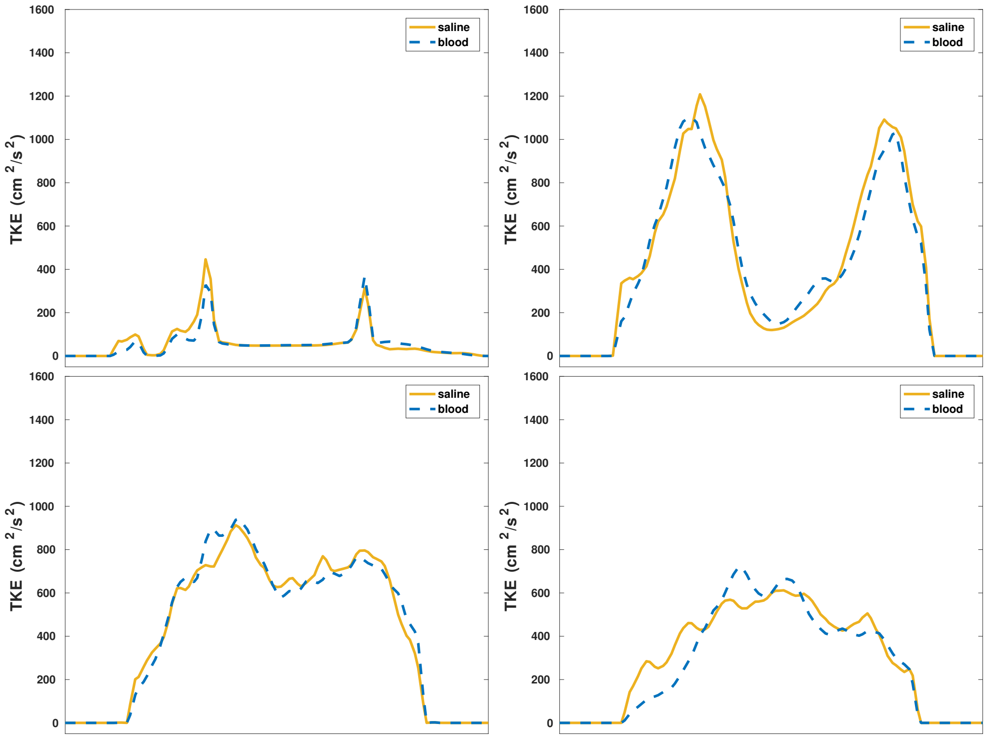


**(b)** 50% maximum steady flow rate

**Figure S21:** (a) Cross-section view of time-averaged turbulence kinetic energy (TKE) profiles at different locations downstream of the valve for the bovine pericardial valve model. We use steady flow at (a) the maximum and (b) 50% of the maximum flow rate. TKE profiles are show at the same locations as indicated in Figures S12 and S13.

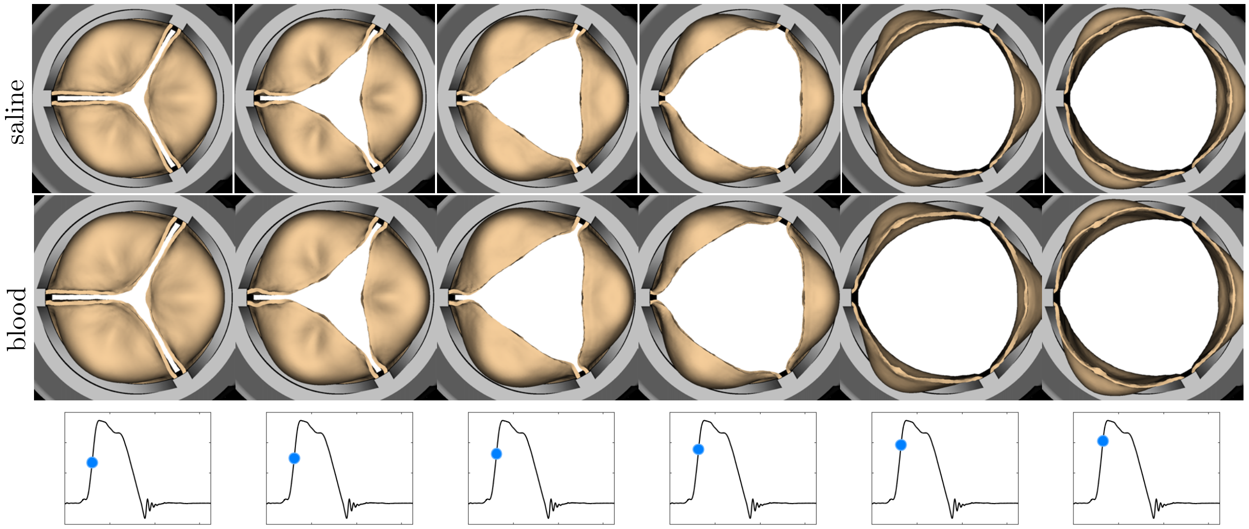


**(a)**

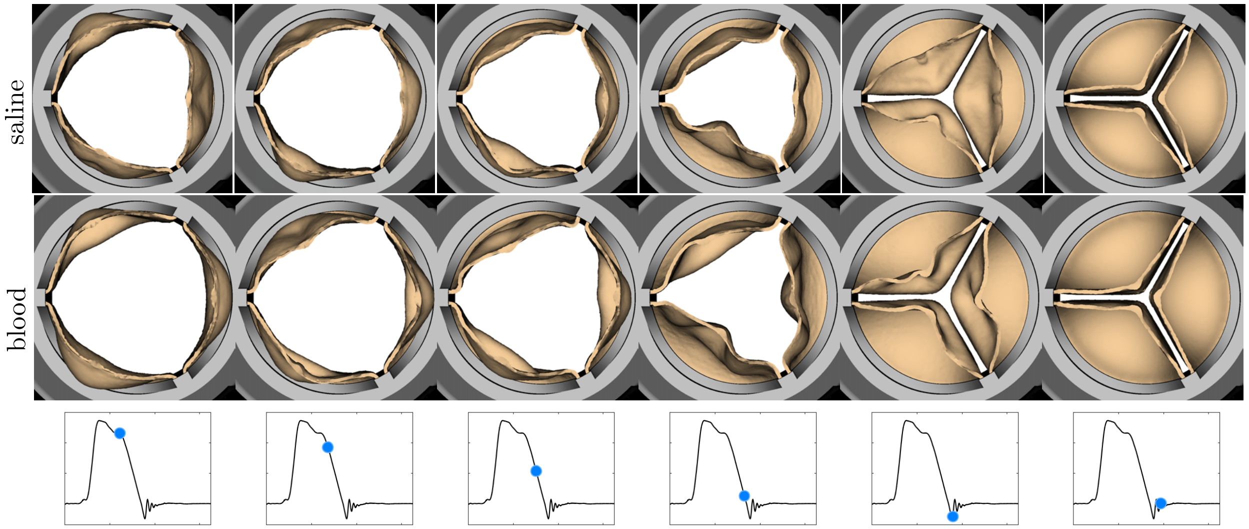


**(b)**

**Figure S22:** Comparison of leaflet kinematics of the porcine aortic valve during (a) valve opening and (b) valve closure between saline and blood analogue. The time increment between frames in panel (a) is 1.92 ms, and the time increment between frames in panel (b) is 9.6 ms.

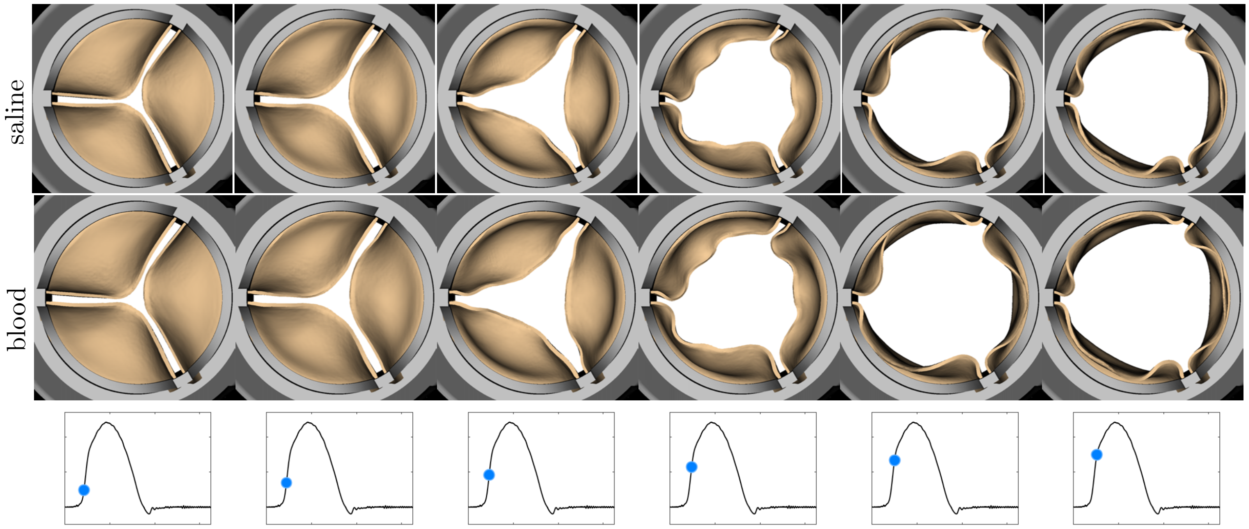


**(a)**

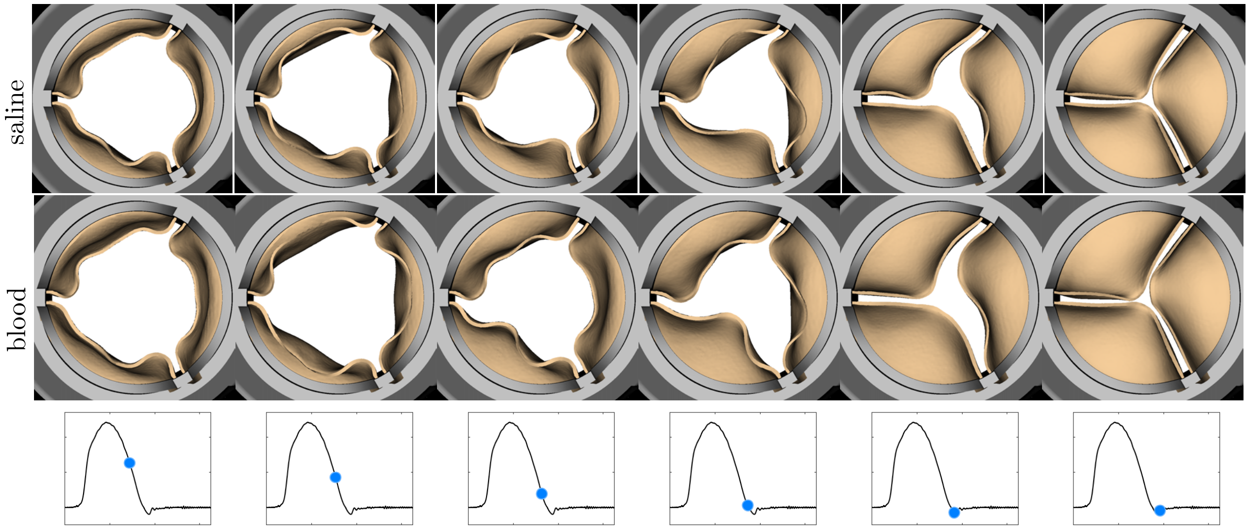


**(b)**

**Figure S23:** Comparison of leaflet kinematics of the bovine pericardial valve during (a) valve opening and (b) valve closure between saline and blood analogue. The time increment between frames in panel (a) is 3.84 ms, and the time increment between frames in panel (b) is 19.2 ms.

# References


[1] K. L. Billiar, M. S. Sacks, Biaxial mechanical properties of the natural and glutaraldehyde treated aortic valve cusp. Part I: experimental results, J Biomech Eng 122 (1) (2000) 23–30.

[2] K. L. Billiar, M. S. Sacks, Biaxial mechanical properties of the native and glutaraldehyde-treated aortic valve cusp. Part II: a structural constitutive model, J Biomech Eng 122 (4) (2000) 327–335.

[3] H. Kim, K. B. Chandran, M. S. Sacks, J. Lu, An experimentally derived stress resultant shell model for heart valve dynamic simulations, Ann Biomed Eng 35 (1) (2007) 30–44.

[4] L. N. Scotten, R. Siegel, Importance of shear in prosthetic valve closure dynamics, J Heart Valve Dis 20 (2011) 664–672.

[5] B. E. Griffith, X. Y. Luo, D. M. McQueen, C. S. Peskin, Simulating the fluid dynamics of natural and prosthetic heart valves using the immersed boundary method, Int J Appl Mech 1 (1) (2009) 137–177.

[6] L. Zhang, A. Gerstenberger, X. Wang, W. K. Liu, Immersed finite element method, Comput Methods Appl Mech Engrg 193 (21–22) (2004) 2051–2067.

[7] W. K. Liu, Y. Liu, D. Farrell, L. Zhang, X. S. Wang, Y. Fukui, N. Patankar, Y. Zhang, C. Bajaj, J. Lee, et al., Immersed finite element method and its applications to biological systems, Comput Methods Appl Mech Engrg 195 (13–16) (2006) 1722–1749.

[8] L. T. Zhang, M. Gay, Immersed finite element method for fluid-structure interactions, J Fluids Struct 23 (6) (2007) 839–857.

[9] X. Wang, L. T. Zhang, Interpolation functions in the immersed boundary and finite element methods, Comput Methods Appl Mech Engrg 45 (4) (2010) 321–334.

[10] X. Wang, C. Wang, L. T. Zhang, Semi-implicit formulation of the immersed finite element method, Comput Methods Appl Mech Engrg 49 (4) (2012) 421–430.

[11] B. E. Griffith, X. Y. Luo, Hybrid finite difference/finite element immersed boundary method, Int J Numer Methods Biomed Eng 33 (11) (2017) e2888.

[12] B. E. Griffith, An accurate and efficient method for the incompressible Navier–Stokes equations using the projection method as a preconditioner, J Comput Phys 228 (20) (2009) 7565–7595.

[13] B. E. Griffith, On the volume conservation of the immersed boundary method, Commun Comput Phys 12 (2) (2012) 401–432.

[14] W. J. Rider, J. A. Greenough, J. R. Kamm, Accurate monotonicity- and extrema-preserving methods through adaptive nonlinear hybridizations, J Comput Phys 225 (2) (2007) 1827–1848.

[15] P. Colella, P. R. Woodward, The piecewise parabolic method (PPM) for gas-dynamical simulations, J Comput Phys 54 (1) (1984) 174–201.

[16] B. Vadala-Roth, S. Acharya, N. A. Patankar, S. Rossi, B. E. Griffith, Stabilization approaches for the hyperelastic immersed boundary method for problems of large-deformation incompressible elasticity, arXiv preprint arXiv:1811.06620.

[17] S. Reese, M. K. Ussner, B. D. Reddy, A new stabilization technique for finite elements in non-linear elasticity, Int J Numer Meth Biomed Engng 44 (1999) 1617–1652.

[18] J. Bonet, A. J. Gil, R. Ortigosa, A computational framework for polyconvex large strain elasticity, Comput Methods Appl Mech Engrg 283 (2015) 1061–1094.
